# Perception of online and face to face microbiology laboratory sessions among medical students and faculty at Arabian Gulf University: a mixed method study

**DOI:** 10.1186/s12909-022-03346-2

**Published:** 2022-05-30

**Authors:** Ronni Mol Joji, Archana Prabu Kumar, Amer Almarabheh, Fazal K Dar, Abdel Halim Deifalla, Yasin Tayem, Abdulrahman Yusuf Ismaeel, Khalid Bindayna, Khaled Saeed Tabbara, Eman Farid, Mohd Shadab, Ali Al Mahmeed, Mohammad Shahid

**Affiliations:** 1 Department of Microbiology, Immunology, and Infectious Diseases, College of Medicine and Medical Sciences, Arabian Gulf University, Manama, Kingdom of Bahrain; 2 Medical Education Unit, College of Medicine and Medical Sciences, Arabian Gulf University, Manama, Kingdom of Bahrain; 3 Department of Family and Community Medicine, College of Medicine and Medical Sciences, Arabian Gulf University, Manama, Kingdom of Bahrain; 4 Department of Anatomy, College of Medicine and Medical Sciences, Arabian Gulf University, Manama, Kingdom of Bahrain; 5 Department of Pharmacology, College of Medicine and Medical Sciences, Arabian Gulf University, Manama, Kingdom of Bahrain

**Keywords:** Online, Face to face, Blended, Focus group discussion

## Abstract

**Background:**

The COVID-19 pandemic has impacted all spheres of society including medical education and healthcare systems. In response to the pandemic, there has been a transition in medical education practice from traditional forms of teaching to online instruction delivery and virtual learning. Effective clinical microbiology education involves a combination of 'hands-on' practical learning and instructional delivery of scientific knowledge. Microbiology practical laboratories are critical learning environments offering 'hands-on' learning experiences that cannot be replicated through online learning. We conducted a mixed-methods study to understand the perception of online and face-to-face microbiology laboratory sessions among the medical students and microbiology faculty at Arabian Gulf University (AGU).

**Methods:**

The study participants were third and fourth-year undergraduate medical students and faculty involved in delivering microbiology labs at AGU. The questionnaire consisted of questions ranging from perceived learning style to attitude towards online delivery of microbiology curriculum. After the questionnaire administration (google form), focus group discussion (FGD) was conducted for students and microbiology faculty separately.

**Results:**

Among 168 students, 50.6% preferred face-to-face lab sessions as compared to 30.4% who preferred online labs, and 51.8% considered online labs to be an essential addition to face-to-face labs. Among the faculty, 85.7% preferred the face-to-face mode of teaching. All the faculty (100%) disagreed that all the microbiology labs teaching should be online. 57.2% considered online labs to be an essential addition to traditional face-to-face labs. Both faculty and students hold that a blended mode of instructional delivery is vital and indispensable for the transfer of skills and knowledge for microbiology students.

**Conclusion:**

The blended mode of delivering microbiology laboratory sessions in medical school is successful and well-received by both students and faculty. Students take the responsibility for furthering their own learning and understanding of concepts. Instructors have also noticed that blending learning strategies also successfully enhances the development of cognitive skills and problem-solving abilities in students. A review of the microbiology lab curriculum is necessary to identify content areas that can be delivered effectively through online, face-to-face lab sessions, or both, supported with appropriate tools and infrastructure.

**Supplementary Information:**

The online version contains supplementary material available at 10.1186/s12909-022-03346-2.

## Introduction

Even before the WHO declaration of COVID-19 a pandemic on March 11, 2020, the closure of all academic institutions had been announced in many regions of the world. Bahrain suspended all academic institutions to curb the spread of COVID-19 infection on 25^th^ February 2020 [1]. By the end of April 2020, 186 countries had implemented nationwide closures, affecting approximately 73.8% of all enrolled students [2]. Since then, the COVID-19 pandemic has continued to wreak havoc on education and healthcare delivery systems globally. The disease has been particularly challenging for medical education as instructors had to deliver lectures, labs, clinical sessions safely while maintaining the integrity of the medical education process [3]. It is fortuitous that over the last few decades, there has been increasing technology integration into medical education and clinical practice (in line with advances in educational and healthcare technologies), such that traditional face-to-face instruction sessions have been implemented in conjunction with the use of digital learning products, online platforms, and electronic consultation [4]. Medical school graduates in the twenty-first century are expected to be technologically savvy, in line with this digital age. There has never been a greater need for educators, students, and clinicians to constantly update their skills, stay current with the changing educational and healthcare environment, and be "digitally knowledgeable" [5].

Many unknowns, from student-driven to staff-driven factors, may determine whether an online learning program succeeds or fails [6]. Clinical Microbiology is an applied study of infectious organisms and their clinic-pathological role in human ailments [7]. Effective clinical microbiology education and learning involves a combination of 'hands-on' practical learning and instructional delivery of requisite scientific knowledge, using instructional approaches that are 'suitable and effective' for delivery of knowledge to large student numbers [8]. Microbiology practical laboratories are critical learning environments in which students acquire hands-on experience and grow their professional expertise. The full range of experience offered in this practicum cannot be delivered through online lectures and library resources [9]. Laboratory practica are a core component of the microbiology curriculum and central to the validation of the training experience [10]. The COVID-19 pandemic has been highly disruptive of the traditional delivery of laboratory training compelling changes in both structure and delivery methods. Technology developments have allowed the switch from face-to-face lab instructions to online and virtual labs. These changes in microbiology lab education are bound to influence students’ experience of learning and learning outcomes. It is, therefore, important to understand students’ perceptions regarding how well their learning needs are met in this learning environment, as well as their perceptions on the efficiency and quality of the new lab experience [10]. Determining outcomes of faculty teaching practices in a course is also important in a variety of contexts, including evaluating courses and assessing the effectiveness of faculty development and student learning outcomes [11].

### Status of face-to-face labs

Face to-face labs provide students with the opportunity to work and learn alongside peers and instructors [10]. It can also lead to increased inner motivation with the advantage of immediate face-to-face feedback [12]. In the US, Hearing et al. reported a 21% reduction in personal labs offered by 70 medical schools between 2002 and 2014 [13]. Contributing factors were the cost of laboratory materials for hands-on experiments, the safety of students, and the availability and salary of instructors [14]. Even though there are several tools available for online teaching [15], none of the online tools can provide hands-on experience comparable to exercises held on-site in the microbiological laboratory. As a result, use of online teaching tools alone does not support the full development of hands-on laboratory skills and associated knowledge; individual on-site laboratory practice is also considered necessary [9].

### Status of online labs

Several studies have reported satisfaction with online laboratory sessions on the basis of cost-effectiveness and convenience [16]. Research studies have suggested that a variety of online digital tools can be used to supplement or even replace laboratory-based microbiological learning. Examples of such digital tools include virtual lab simulation [10] and video lectures [15]. The faculty can provide instruction, and students can access the instructional sessions from any location, eliminating the cost of laboratory space and materials and travel cost and time [17].

### Status of blended learning

Blended learning combines online and in-person learning; this approach to learning factors in financial and faculty constraints as well as students’ learning priorities or prerequisites [10]. Assigning online labs to students prior to lab exercises or tests appears to boosts their confidence and motivation for learning [18]. Even though many microbiology educationalists remain skeptical of the use of dry/ online labs, evidence suggests that when used in conjunction with wet labs, dry labs can improve student learning. Delivery cost for microbiology practical classes have also been reduced in several university departments in both the United Kingdom and the United States [14].

There remains a paucity of published studies on the preferred mode of learning and teaching for microbiology laboratory sessions in medical schools. Hence, we aimed to conduct a mixed-methods study which might help to delineate microbiology learning needs and how well they are being met and help improve student learning outcomes. This article discusses the perception of microbiology laboratory sessions among the medical students and microbiology faculty at the Arabian Gulf University (AGU).

## Materials and methods

The study was approved by the Research and Ethics Committee, CMMS, Arabian Gulf University (E049-PI-4/21). The participants were third and fourth-year undergraduate medical students, and faculty involved in delivering microbiology labs to undergraduate medical students at AGU. We selected third and fourth-year undergraduate medical students as they had the opportunity to experience both, face to face and online (ZOOM platform) mode of learning microbiology lab sessions. According to the approved protocol, a questionnaire was prepared for the students which was adapted from Salter and Gardner [12]. The faculty questionnaire was developed from literature search and opinion from educational and subject experts. All students (year 3 & year 4) and microbiology faculty were asked to complete the survey while reflecting on their own experience in microbiology labs (face to face & online). The survey consisted of questions ranging from perceived learning style to attitude towards online delivery of microbiology curriculum.

### Microbiology curriculum at AGU

At AGU, we follow Problem Based Learning (PBL) curriculum. There are a total of 9 units (3 units each year) with 93 problems in Phase II (year 2, year 3, year 4). The problems are designed in accordance with regional and global clinical scenarios. The microbiology laboratory sessions (MLS) are designed to correspond to the learning outcomes of PBL sessions. The MLS focuses on the student’s ability to demonstrate pre-specified microbiology laboratory skills, identify, and differentiate common pathogens based on microscopical examination (wet mount, staining), culture (artificial culture media, cell lines), serological and molecular methods, evaluate and interpret the laboratory results and relate these laboratory test results with the clinical scenarios. The MLS are divided into wet labs and dry labs. The students are divided into groups of 20-25 each and the sessions lasts about 2-3 hours. All the students are given the opportunity to execute a specific lab skill under supervision.

For this questionnaire-based study, lab sessions were assessed in two formats: 1) face-to-face lab sessions before the closure of the institution and 2) online lab sessions after the closure of the institution on February 26^th^, 2020, when all the educational institutions were officially closed in Bahrain and online teaching was initiated.

The questionnaire was divided into three parts (Additional files 1 and 2):

- Part 1 related to demographic information.
- Part 2 related to perception of online and face to face (f2f) microbiology lab sessions to which the students and faculty responded using Likert scale.
- Part 3 related to their preferences for, and perceptions of, face to face lab sessions, online lab sessions or a blended form.

The questionnaire contained four items for face-to-face perception and eight items for online perception. Students and faculty selected their responses from a five-point Likert scale from “strongly agree” through “strongly disagree” options. Students were asked about the effectiveness of the current pandemic-induced format for lab delivery and their preferences in regard to online and face-to-face lab sessions. They were also asked about the quality of interactions and strategies to enhance learning under the new educational environment. The questionnaire was administered through google form and the link was shared through emails, and WhatsApp.

After the questionnaire administration, separate focus group discussion (FCD) sessions were conducted for students and microbiology faculty. The semi-structured interview guide for FCD was validated by content and education experts. There were three focus groups: 1) Faculty group 2) Year 3 students’ group and 3) Year 4 students’ group. The FCDs were conducted through ZOOM platform. All the sessions were recorded with the knowledge and consent of the participants. Informed consent was obtained from the study participants before the FCDs. The discussions were transcribed into word document verbatim.

The sample size for the study was:

- Number of students in year 3 (*n*= 170), responders (*n*=86, 50.5%)
- Number of students in year 4 (*n*= 184), responders (*n*=82, 44.5%)
- Number of faculty (*n*=10), participants (*n*=7, 70%)
- Total responders 175: Students (*n*=168) and Faculty (*n*=7)

### Data collection

Data was gathered over a three-month period from year 3 and year 4 undergraduate medical students and faculty at AGU. Medical student and faculty participation in the study was completely voluntary. No personal information was collected in the study and all responses were anonymous. Medical student participation did not influence grading in the course.

### Data analysis

The data from the questionnaire was imported using Statistical Package for the Social Sciences (SPSS Version 27). Variables were presented as counts and percentages or as means and standard deviations where applicable. The validity and reliability of the questionnaire was tested using appropriate statistical methods (Cronbach’s alpha coefficient, Face validity). A cluster bar graph was plotted to represent the distribution of the two qualitative variables. Independent sample t test was used to compare the mean of quantitative variables between groups, and the Chi-Square test was used to compare the proportions between categorical variables. A *p*-value of 0.05 or less was considered as statistically significant.

The data collected from the focus group discussions were transcribed and analyzed using thematic analysis. The approach adopted for the analysis was the inductive approach in which codes obtained from the data were directed by the content of the data and were not based on any preexisting coding frame or preconceptions. Both coding and theme development were done using Microsoft Excel. Raw data was logged into an Excel spreadsheet and sections of text and ideas that appeared repetitively in the data color coded. Such codes were revisited and labeled as they relate to a theme. In order to strengthen accuracy in the analytic process, themes were rechecked against codes and raw data set, creating a map that linked raw data, codes, and the themes that emerged from the data.

## Results

### Demographics and Trends in attendance for online practical sessions

For the 168 student respondents, the mean age was 21.35±2.16 years, mostly females (72.6%).

The mean age for year 3 students was 20.93 (Mean=20.96 for male and 20.92 for female). The mean age for Year 4 students was 21.78 (Mean=21.41 for male and 21.92 for female). Among faculty responders (*n*=7) females were 28.5% (*n*=2). Mean age for male faculty was 53.8 and for female was 51.5.

Data was compiled to understand the trends relating to student and faculty attendance in online practical sessions. Ninety minutes was the allotted time for the online practical sessions at AGU. Table 1 shows the trend in students’ attendance rate over 3 hours span.

The rates for attendance at online practical sessions was highest for year 3 (57%) compared to year 4 students (56.1%). Majority of the respondents attend online practical sessions for 60-90 minutes (56.5%) compared to attendance for 30-60 minutes (38.7%) and attendance for less than 30 minutes (4.8%). The results of the chi-square test showed (Table 1) that there was no association between attendance of year 3 and year 4 students for online practice sessions (χ2=0.635, *p*=0.727). Among the faculty, 57.1% (*n*=4) spent 30-60 minutes and 42.9% (*n*=3) spent 60-90 minutes on each online practical teaching.

**Table 1:** Association between students’ attendance for online practical sessions

Students	Less than 30 minutes n (%)	30-60 minutes n (%)	60-90 minutes n (%)	χ^2^ Statistics (df)	*P* value
Year 3	3 (3.5)	34 (39.5)	49 (57)	0.638	0.727
Year 4	5 (6.1)	31 (37.8)	46 (56.1)		
Total	8 (4.8)	65 (38.7)	95 (56.5)		

### Preferred student location for online study: On-campus (lab) and Off-campus (home/hostel/others)

Students studied the online lab sessions either on campus, off campus or a combination of both. The results indicated that most of the respondents (Year 3 and Year 4 students) attend online lab sessions off-campus (79.2%) compared to attendance for a combination of both on-campus and off-campus (18.5%) and attendance on campus (2.4%) (Table 2). The results of chi-square test showed that there is no association between the preference of location among year 3 and year 4 students for online study.

**Table 2:** Location and number of students attending online lab sessions

	**On Campus n (%)**	**Off Campus n (%)**	**Both n (%)**	χ^2^ Statistics (df)	*P* value
Year 3	2 (1.2)	68 (40.5)	16 (9.5)	0.005 (2)	0.998
Year 4	2 (1.2)	65 (38.7)	15 (8.9)		
Total	4 (2.4)	133 (79.2)	31 (18.5)		

### Preferred faculty location for online lab teaching

All the faculty (*n*=7, 100%) were conducting online lab sessions on campus.

### Student perception about face to face and online microbiology lab sessions

Of the 168 student’s respondents, 50.6% preferred face-to-face lab sessions as compared to 30.4% who preferred online sessions. 60% of all respondents favored face-to-face sessions for better understanding of the course while 51.8% considered online labs to be a useful addition to traditional face-to-face labs (Table 3).

**Table 3:** Likert scale item responses for face to face and online lab sessions by all the students

Item	Description	Percentage of respondents (*n*=168)						
		SD	DA	Total	N	A	SA	Total
	**Students’ perception of the face-to-face microbiology lab sessions**							
1	The face-to-face labs were enjoyable	7.7	9.5	17.2	23.2	30.4	29.2	59.6
2	I preferred the face-to-face	6.0	11.3	17.3	32.1	24.4	26.2	**50.6**
3	Face-to-face labs enhanced my understanding of the course	5.4	12.5	17.9	22.0	32.7	27.4	**60.1**
4	All labs should be face-to-face	9.5	25.0	34.5	31.0	13.1	21.4	34.5
	**Students’ perception of the online microbiology lab sessions**							
1	The online labs were enjoyable.	18.5	17.3	35.8	26.8	24.4	13.1	37.5
2	I preferred the online labs.	20.2	19.0	39.2	30.4	16.7	13.7	30.4
3	Online labs enhanced my understanding of the course	19.6	11.9	31.5	28.6	28.6	11.3	39.9
4	The different types of interaction (animations etc.) in the online labs improved my learning	16.1	11.9	28.0	33.9	24.4	13.7	38.1
5	I found it difficult to follow the flow and meaning of the subject material in the online labs	15.5	26.2	41.7	31.0	13.7	13.7	27.4
6	I consider the online labs to be a useful addition to traditional face-to-face labs	14.9	13.1	28.0	20.2	23.2	28.6	**51.8**
7	Technical enablement (internet/software/hardware) of online sessions was satisfactory	9.5	10.7	20.2	29.8	31.5	18.5	50.0
8	All labs should be online	30.4	21.4	51.8	29.8	6.5	11.9	18.4

*SD* Strongly disagree, *DA* Disagree, *N* Neutral, *A*: Agree, *SA* Strongly agree

Of the 7 faculty/ instructors, 85.7% (*n*=6) preferred face-to-face mode of teaching with the same percentage and majority (85.7%, *n*=6) believing that face-to-face sessions enhanced students understanding of the course. They all agreed that not all lab sessions should be entirely online; though; over half (57.2%) considered online labs to be an essential addition to traditional face-to-face labs (Table 4).

**Table 4:** Likert scale item responses for face to face and online lab sessions by the faculty

Item	Description	Percentage of respondents (*n*=7)						
		SD	DA	Total	N	A	SA	Total
	**Faculty perception of the face-to-face microbiology lab sessions**							
1	The face-to-face lab teachings were enjoyable.	0	0	0	14.3	0	85.7	85.7
2	I preferred the face-to-face lab teaching.	0	0	0	14.3	14.3	71.4	**85.7**
3	In my opinion face to face labs enhanced students understanding of the course.	0	14.3	14.3	0	0	85.7	**85.7**
4	In my opinion all the lab sessions should be face to face.	0	0	0	28.6	28.6	42.9	71.5
	**Faculty perception of the online microbiology lab sessions**							
1	The online lab teachings were enjoyable.	0	0	0	42.9	57.1	0	57.1
2	I preferred the online lab teaching.	0	42.9	**42.9**	57.1	0	0	0
3	In my opinion online labs enhanced students understanding of the course.	0	28.6	28.6	57.1	0	0	0
4	In my opinion the different types of interaction (animations etc.) in the online labs improved students learning.	0	14.3	14.3	42.9	42.9	0	42.9
5	In my opinion the students found it difficult to follow the flow and meaning of the subject material in the online labs.	0	28.6	28.6	28.6	42.9	0	42.9
6	I consider the online labs to be an essential addition to traditional face to face labs.	0	14.3	14.3	28.6	28.6	28.6	**57.2**
7	Technical enablement (internet/software/hardware) of online sessions was satisfactory.	0	14.3	14.3	14.3	42.9	28.6	71.5
8	In my opinion all the lab sessions should be online.	42.9	75.1	100	0	0	0	0

*SD* Strongly disagree, *DA* Disagree, *N*: Neutral, *A* Agree, *SA* Strongly agree

### Opinion on preferred mode of learning and teaching

**Fig. 1:**
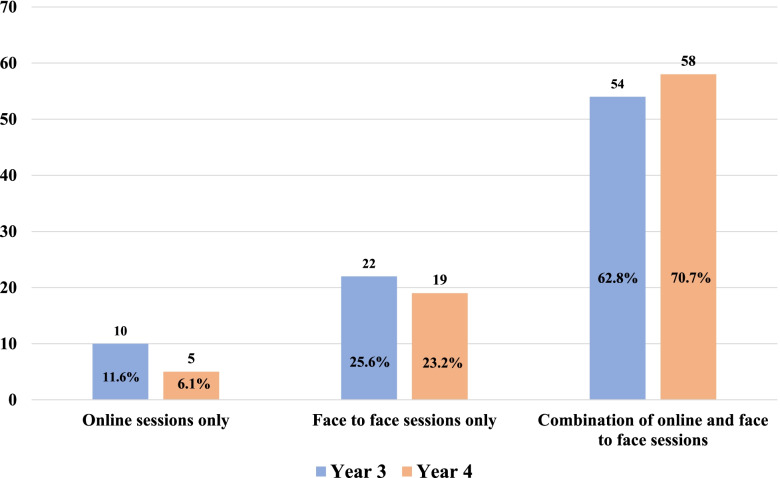
Preferred mode of learning among students

Blended lab sessions using both modes of delivery were preferred by year 3 (62.8%) and year 4 (70.7%) students (Fig. 1). The results of the chi-square test showed that there is no association between the preference of year 3 students and year 4 students towards the mode of learning microbiology lab sessions (χ2=1.935, df=2, *p*=0.380). All the faculty (100%, *n*=7) preferred a blended mode of teaching microbiology lab sessions.

### Comparative analysis of AGU lab curriculum with American Society for Microbiology (ASM) lab curriculum guidelines

In the pre-COVID period, microbiological lab sessions at AGU were conducted face to face. However, during the COVID-19 pandemic, lab sessions were held via the ZOOM platform. We compared the AGU lab curriculum to the internationally recognized ASM curriculum recommendations for undergraduate microbiology education. According to the ASM microbiology lab skills criteria, the AGU microbiology lab objectives were met (Table 5). At AGU, most of the year 3 and 4 students and faculty members chose a blended learning and teaching environment (Table 5).

**Table 5:** Comparative analysis of Microbiology lab curriculum

**ASM Objectives Of Undergraduate Microbiology Education (Part 2: Microbiology Lab Skills)**	**AGU Microbiology Lab Objectives**	**Faculty preference (*n*=7)**			**Year 3 students’ preference (*n*=86)**			**Year 4 students’ preference (*n*=82)**		
		**Online**	**Face to Face**	**Blended**	**Online**	**Face to Face**	**Blended**	**Online**	**Face to Face**	**Blended**
1 Properly prepare and view specimens for examination using microscopy (bright field and, if possible, phase contrast).	**✓ **	23%	34%	**44%**	21%	35%	**44%**	23%	34%	**44%**
2 Use pure culture and selective techniques to enrich for and isolate microorganisms.	**✓**	26%	28%	**46%**	20%	35%	**45%**	22%	34%	**44%**
3 Use appropriate methods to identify microorganisms (media-based, molecular and serological).	**✓**	28%	26%	**47%**	20%	35%	**45%**	23%	34%	**44%**
4 Estimate the number of microorganisms in a sample (using, for example, direct count, viable plate count, and spectrophotometric methods).	**✓**	0%	29%	**71%**	20%	36%	**44%**	22%	33%	**45%**
5 Use appropriate microbiological and molecular lab equipment and methods.	**✓**	30%	24%	**46%**	20%	35%	**44%**	23%	34%	**44%**
6 Practice safe microbiology, using appropriate protective and emergency procedures.	**✓**	31%	24%	**45%**	19%	33%	**48%**	22%	34%	**46%**
7 Document and report on experimental protocols, results, and conclusions.	**✓**	56%	14%	**30%**	26%	33%	**41%**	27%	30%	**45%**

### Findings from Thematic analysis of the data from the Focus Group Discussion (FGD)

The analysis yielded six main learning/teaching themes and sub-themes as shown in Table 6.

**Table 6:** Thematic outcomes of FGD

**Thematic area (6)**	**Sub themes (14)**	**Codes (176)**
Virtual sessions	Educational technologies for virtual lab sessions	52
	Pros of online mode	
	Cons of online sessions	
Face-to-face sessions	Physical presence	38
	Pros of online mode	
	Cons of online sessions	
Dry lab	Theory-based, concepts	11
Wet Lab	Practical demonstration by instructors	20
	Hands-on learning by students	
Curriculum	Online suitability	31
	Face to face suitability	
	Blended learning	
Lessons learned	Areas for refinement	24
	Emergent student, institutional and department needs	

The six main themes that emerged from the study are as follows:

#### Pros and cons of online microbiology lab sessions

The advantages of online lab sessions are: easy access to sessions from any location; use of tools such as PowerPoints, images, case studies, clinical cases, and videos that facilitate effective delivery of theoretical content; sessions can be recorded allowing students to revisit the session and deepen learning; instructors can also review recorded sessions for evaluative or other purposes as needed; elimination of lab management challenges related to large groups; and better time management (elimination of time spent waiting for shared lab facilities to become available, scheduled time frames for online lessons).

The drawbacks to online lab sessions are that both teaching and learning are less adaptable for wet labs compared to dry labs; challenges with evaluating student learning; deficits with regard to acquisition of cognitive skills since students cannot engage in hands-on lab activities or learn how to use actual lab equipment; poor feedback compared to face-to-face sessions where there is direct communication, including emotional cues; and technological glitches that may impede teaching and learning. Inconsistency in level of student engagement in online sessions was also identified as a drawback by both faculty and students. As observed by a Year 3 student “So, there is no problem with gaining information, but the problem is for staying focused I think, during the session”.

#### Pros and cons of face-to-face microbiology lab sessions

The pedagogical strategies used in face-to-face lab sessions are already well-established; instructors are skilled in these practices leading to efficient teaching and assessment; students are able to gain practical and cognitive skills through hands-on participation; socially and mentally beneficial physical interaction takes place in face-to-face lab settings; effective bi-directional feedback; and higher retention of information by students. This mode of delivery of lab sessions is also effective for both wet and dry labs.

The drawbacks to face-to-face microbiology lab sessions are indirect rather than direct: less flexibility in the teaching and learning process such that the student and teachers must be in the physical location for the lab session; time constraints such as travel time to lab location; some waste of material resources; and tendency to repeat rather than innovate with technical methodologies.

#### Use of online modes for dry lab sessions/theory-based sessions

Online laboratory sessions are effective for delivering dry labs which are theory-based microbiology lab sessions in which concepts are explored and conceptual knowledge acquired. Assessment of students’ learning regarding such theoretical aspects can also be done online.

#### Use of face-to-face modes for wet lab sessions/ practica

Online lab sessions do not support the delivery of wet lab which are lab sessions that require and involve demonstration of some experimentation to students and sessions that require students to learn how to use lab tools such as the microscope for slide preparation and examinations or diagnostic procedures from cultures to immunodiagnostics. For such wet lab sessions, face-to-face sessions are required. Evaluation of students’ practical skills and their learning is also done during such face-to-face sessions.

#### Curriculum development based on blended learning

Blended learning, which is strategic combination of both online lab sessions and face-to-face lab sessions in the delivery of the microbiology lab curriculum, emerged as the most efficient way to deliver this course. Certain procedures can be effectively learned using a combination of online and face-to-face instructions; some activities can be effectively conducted in the online mode eliminating the need for physical presence, and others can only be conducted only in a physical environment. For instance, sample handling requires physical presence for the processing activities, but reporting may be done online. As stated by one of the participants, “Sample processing... it's not going to get theoretical. It must be done face to face. They have to handle the specimen”. A blended learning curriculum takes into consideration the possibility, benefits, and advantages of teaching and learning processes in either the online, face-to-face mode, or both as appropriate.

Thus, the emergence of blended learning as a critical strategy for the delivery of microbiology lab is based on both the requirements for microbiology education and the pre-COVID-19 trend toward technology integration. The adoption of online learning in response to the pandemic reinforced this phenomenon. Post-COVID-19, if the virus is controlled, technological adoptions made during the pandemic will not only remain but will likely accelerate as more technological innovations are developed and society continues to exploit the benefits of remote education. This scenario creates the need for a curriculum review to determine a blended learning curriculum that is most effective for microbiology education and a best fit for the institution.

#### Development and refinement of tools to support online delivery of lab sessions

The institution is growing and evolving in its use of online lab sessions during the COVID-19 pandemic. Constant review of the teaching strategies, student outcomes, and formulating of test curricula has helped optimize transfer of knowledge during the pandemic, bringing cost benefits for the department and institution. Infrastructural challenges exist for effective delivery of blended learning. Tools such as videos and images must be further developed. A core of IT technical support team with specialized training must be developed, and training workshops implemented for both faculty and students on all relevant aspects.

## Discussion

This study investigates student and faculty perception of the different modes of learning and teaching microbiology laboratory sessions at AGU. Previous research has mainly focused on the impact of online and in-person labs, with little emphasis on student [10] and faculty feedback. Using a survey and focus group discussions, we identified the most important variables in perceptions of learning and teaching in the new learning environment as being flexibility, interaction, understanding, and learning and teaching preference.

### Face-to-face lab preferred for microbiology lab skills

In our findings, traditional delivery of microbiology lab sessions has been based on the face-to-face model. Both faculty and students hold that this mode of delivery continues to remain vital and indispensable for the transfer of skills and knowledge for students. Certain aspects of laboratory training can only be done effectively using face-to-face lab sessions, specifically, wet labs. These lab sessions are associated with gain in cognitive and practical skills, increased retention of information, and quality interaction between faculty and students. This was consistent with Brockman et al. who found that 90% of the students, preferred face-to-face lab sessions [10]. Similarly, Salter and Gardner also noted that students prefer face to face labs to online labs [12]. Another study by Kay et al. also reported 50% of the students preferred both face to face and online lab sessions [19]. Our observations, combined with those of other researchers, support the notion that most students respond better to face-to-face instruction [20]. Especially in a practical session, face-to-face teaching allows faculty to tailor teaching methods to the specific needs of students instantaneously and to answer questions from the students directly [21]. From the constructivist perspective, active learning allows students to teach each other and to build on understanding on frameworks of microbiological knowledge. The pandemic-induced transition from face-to-face to online teaching has created significant challenge for students in terms of acquiring skills, engaging students and encouraging active learning [22].

### Online lab preferred for theoretical sessions

Despite the overwhelming number of students opting for face-to-face lab sessions, our data indicate a minimal trend in which students (30.4%) prefer online learning as the ideal format for microbiology labs. Among the faculty, none opted for online microbiology labs except for delivering theory-based knowledge and case discussions. There are also barriers to online teaching and learning, particularly in large-enrollment courses, although such barriers also present opportunities to innovate and improve align our curricula with the current educational environment [23].

The major focus among microbiology educators remains the provision of tried and tested face-to-face microbiology practical sessions. Because the acquisition of core "hands-on" skills is crucial in many science and fine arts courses, finding appropriate instructional strategies during the pandemic has been an urgent task [9]. Hands-on lab skills and experience that encompass best practices for handling and observing microorganisms are included in the American Society for Microbiology (ASM) curricular guidelines for undergraduate microbiology [24]. These skills include following aseptic procedures, proper handling of tools, reagents, and equipment, and microbe manipulation. These abilities cannot be fully built using videos or simulations. Students require laboratory-based practice and feedback to improve technical skills such as sequential feedback on technical methods, use of equipment, and interpretation of results [9]. Research on preference for online teaching has yielded mixed results. For instance, students response in Salter and Gardners’ [12] study indicated a preference for one-to-one guidance from educators, a potential advantage of face-to-face sessions over online sessions, which is also supported by our findings. However, these findings were not in agreement with the findings by Polly et al. where undergraduate students felt that online lab sessions were the same or better than face to face experience [25]. Kay et al. also suggested that online labs were more helpful than face to face labs [19]. In our study, the potential advantage that students found in online lab sessions were its effectiveness for theoretical sessions, its advantage of flexibility, elimination of time constraints, and effectiveness in the transfer of knowledge using online tools.

### Blended mode of delivery of microbiology laboratory sessions

Blended learning is a teaching method that combines online self-learning with classroom instruction. When well-designed, blended learning courses in medicine can improve students self-learning, insight, and problem-solving skills, ultimately increasing the quality of learning [26]. Blended learning is a promising approach for delivering diverse curricula content effectively as it allows the morphing of effective instructional practices [27]. In the present study, AGU microbiology lab objectives fulfilled the ASM microbiology laboratory recommendations and both faculty and students identified blended learning as being an essential approach to maximize learning in lab sessions. Our findings agree with those of Chen et al, who found most of their students (63.01%) preferred blended laboratory courses [26] and with Salter and Gardner who also reported on the student’s preference for blended learning [12]. A study by Lu proposed the creation of a blended learning environment to enhance analytical reasoning, noting that students were satisfied with this mode of learning and considered the blended learning environment important for critical thinking [28]. Another study by Masiello et al on a microbiology course noted that the students expressed favorable perceptions toward blended mode of learning and derive long-term benefit from its use [29]. Similar results were reported by Sancho et al [30]. Blended sessions represent a fundamental rethinking and reorganization of the teaching and learning dynamic, beginning with the identification of context - specific needs and potential outcomes [30]. In medical education, the online component of blended courses consist of high-quality online courses aimed at the effective delivery of basic medical courses and improving students' study skills and motivation [26].

The limitations of this study may provide a basis for future research on laboratory perceptions in medical schools. Our study included year 3 and year 4 medical students (phase II) in a six-year medical undergraduate programme, and the response rate was only 50%. The faculty sample size was also small. One of the drawbacks of FGD is that study participants may not express their honest and personal opinions about the subject at hand. They may be reluctant to voice their opinions especially if their opinions differ from those of another participant. Future research addressing these limitations may be important for providing additional insight into perceptions regarding microbiology lab sessions under the current educational environment.

In conclusion, blended mode of delivering microbiology laboratory sessions in medical school are successful and well-received by both students and faculty. Students take the responsibility of self-learning which improves their abilities to think critically while acquiring a deeper understanding of concepts. Instructors noted that these innovative sessions successfully enhance the development of cognition and problem-solving abilities in students. Instructors also became more effective in the delivery of knowledge when students engage in self-directed learning rather than passing theoretical knowledge. Review of microbiology lab curriculum is necessary to identify content areas that can be delivered effectively through online modalities, face-to-face lab sessions, and blended formats. Appropriate tools and infrastructure aligned with the microbiology lab curriculum must be developed in support of online formats including videos, images, specialized training tools, and case studies since this is still an emerging pedagogical format.

## Supplementary Information

**Additional file 1.**

**Additional file 2.**

**Additional file 3.**

**Additional file 4.**

**Additional file 5.**

**Additional file 6.**

**Additional file 7.**

## Data Availability

All data generated or analysed during this study are included in this published article [and its supplementary information files].
