# Supplementary material for: Perception of online and face to face microbiology laboratory sessions among medical students and faculty at Arabian Gulf University: a mixed method study
Source: BMC Med Educ. 2022 May 30;22:411. doi: 10.1186/s12909-022-03346-2 (PMC9149330; doi:10.1186/s12909-022-03346-2)
Supplement: Supplementary file 2 — Additional file 2. [file 12909_2022_3346_MOESM2_ESM.pdf]

# FACULTY RESPONSES

| Timestamp | Consent to participate in this survey | Gender | Year of teaching | Amount of time did you spend on each online practical teaching | Where did you work on the online lab sessions? | The f2f lab teachings were enjoyable | I preferred the f2f lab teaching | In my opinion f2f labs enhanced students understanding of the course | In my opinion all the lab sessions should be f2f | The online lab teachings were enjoyable | [I preferred the online lab teaching | In my opinion online labs enhanced students understanding of the course | In my opinion the different types of interaction (animations etc.) in the online labs improved students learning | In my opinion the students found it difficult to follow the flow and meaning of the subject material in the online labs | I consider the online labs to be an essential addition to traditional f2f labs | Technical enablement (internet/software/hardware) of online sessions was satisfactory | In my opinion all the lab sessions should be online | The best option to deliver microbiology lab sessions would be through one of the following | Give reasons for your response to the above question |
|---|---|---|---|---|---|---|---|---|---|---|---|---|---|---|---|---|---|---|---|
| 2021/05/05 9:23:31 am GMT+3 | Yes | Female | Year 2;Year 3;Year4 | 60-90 minutes | On Campus | SA | A | SA | A | A | N | A | N | N | SA | SA | D | COMBINATION of online and face to face sessions | some lab sessions needs skills which cannot be achieved by online |
| 2021/05/10 2:51:25 pm GMT+3 | Yes | Male | Year 2;Year 3;Year4 | 30-60 minutes | On Campus | SA | SA | SA | SA | N | N | N | N | A | A | N | D | COMBINATION of online and face to face sessions | Handling actual patient's specimen cope with clinical practice of the future being clinician |
| 2021/05/12 9:43:21 am GMT+3 | Yes | Male | Year 3;Year4 | 60-90 minutes | On Campus | SA | SA | SA | A | A | D | N | A | D | SA | SA | D | COMBINATION of online and face to face sessions | Some laboratory sessions require hands-on sessions and thus require f2f sessions. On the other hand, some labs are given as dry labs so online mode of delivery would be more effective and beneficial where the concepts can be discussed effectively through PowerPoint presentations with some videos (if required). |
| 2021/05/12 11:30:58 am GMT+3 | Yes | Male | Year 2 | 60-90 minutes | On Campus | N | N | D | N | N | N | D | D | A | N | D | D | COMBINATION of online and face to face sessions | Mainly, we should do f2f lab. sessions but sometimes, if subject allows, we can go for online teaching. |
| 2021/05/18 9:52:59 am GMT+3 | Yes | Male | Year 2;Year 3;Year4 | 30-60 minutes | On Campus | SA | SA | SA | SA | A | N | N | A | A | D | A | SD | COMBINATION of online and face to face sessions | the lab should be f2f and additional materials and films should be recorded online |
| 2021/05/23 12:19:26 pm GMT+3 | Yes | Male | Year4 | 30-60 minutes | On Campus | SA | SA | SA | SA | A | D | N | A | D | N | A | SD | COMBINATION of online and face to face sessions | f to f important for practical training & online for conceptual |
| 2021/05/24 6:50:13 am GMT+3 | Yes | Female | Year 2;Year 3;Year4 | 30-60 minutes | On Campus | SA | SA | SA | N | N | D | D | N | N | A | A | SD | COMBINATION of online and face to face sessions | some topics need online explanations but to see life eg under microscope is an important lab skill |

Footnote: SD - Strongly Disagree, D - Disagree, N - Neutral, A - Agree, SA - Strongly Agree
