# Supplementary material for: Perception of online and face to face microbiology laboratory sessions among medical students and faculty at Arabian Gulf University: a mixed method study
Source: BMC Med Educ. 2022 May 30;22:411. doi: 10.1186/s12909-022-03346-2 (PMC9149330; doi:10.1186/s12909-022-03346-2)
Supplement: Supplementary file 3 — Additional file 3. [file 12909_2022_3346_MOESM3_ESM.pdf]

Female Speaker 2:

So, very good morning and welcome to our focus group discussion today. This focus group discussion is conducted as part of this study, titled *The Perception of Online and Face-to-Face Microbiology Laboratory Sessions and on Medical Students and Faculty at Arabian Gulf University*. I am one of the investigators of this project and we have with us, Dr. Archana from the department of medical education who is also a co-investigator in this project. So, thank you all for giving your informed consent for taking part in this focused group discussion. And please feel free to share your views about your experiences with online as well as face-to-face microbiology laboratory sessions at AGU. This study is approved by research and ethics committee AGU number E049BI4/21. The (00:10:00) discussion will be video recorded to ensure that we did not miss any of your valuable comments. It would last for around 30 minutes and all the contents of the focus group discussion will be maintained confidential, and your identity will not be revealed in any manner. The transcribed files will be kept in a secure location and will be destroyed after completion of the study. So please feel free express your thoughts and we will try our level best to take your valuable comments into account for our future microbiology laboratory sessions. Thank you all once a week. So, let's begin. Dr. Archana please go ahead.

Dr. Archana:

Thank you, Ronnie. Thank you, department of microbiology for giving us this opportunity and education is a dynamic process. We have to learn from the past to correct the present and the future. We all know that Covid-19 we had to shift to online session and definitely we had some challenges to face and it's not hundred percent ideal as of now. So, we would like to take your perceptions, your feedback and suggestions and we'll try to make it as good as possible in future. So let us reflect upon our experience and let us take this forward. We have around eight questions to discuss. The first three questions are mainly focusing on online. I mean, first three questions are based on online microbiology lab sessions, and then we will talk about face-to-face lab session and the last two questions are in general we are going to discuss how it can be made better. So let me start with the first question. Can you please describe your experience teaching online microbiology lab session? Your experience on online microbiology lab session? Anyone can start.

Male Speaker 1:

Well, I can start with zero. Can you hear me?

Dr. Archana:

Yes, please proceed.

Male Speaker 1:

I must confess I have not been involved in this online lab sessions, so I'm sort of as somebody who's watching from the sidelines, so to speak. So, anything I have to say is really based on my own observations in terms of other modes of learning online but here, including clinical teaching as well so but here is something which has to do with the lab, which include skills, certain exercise of skills. So, whatever thoughts I have would be based on sort of a broader aspect of using online media, online techniques to sort of have an exchange process because this is a lab session, particularly are not just one way thing that you are telling the students to do something.

You are also watching them do something. So, it's just a two-way process and the evaluation is also based on that. On whether you have been successful in imparting whatever skills that you wish to impart, whatever specific skills that you wish to impart, and whether the students have achieved a certain minimum level of skills in that particular aspect. So, it's compartmentalized in the sense that although there's an overall look at the student's ability to make use of whatever tools he or she has at the disposal in the lab as a microscope or a scalpel or whatever. And then you are really making an observation on how the student attends to that particular task of looking down a microscope and describing what he or she sees under the microscope, or does a dissection and displays shows bits and pieces of a mouse or rat or a limb or whatever. So, I think that experience of online exchange between the teacher and the student is something which I think we've had a little bit of experience in terms of clinical teaching and observing students attend to a case and so on. But as I said, I have not really attended any lab session as such. So, my observations on that will be very, very limited in terms of what benefit was accrued by the students and indeed by the course itself, because the course itself benefits, if it gets this kind of feedback from (00:15:00) whether there's a correction element required, or maybe there is a lot of redundancy in what we are giving and we can get rid of that. And while at it maybe I can also put in another thought and that is it's also an opportunity because nothing to do with the online learning, just because of the rapid increase in the amount of information that we

have, the amount of research productivity that we have particularly in these applied sciences, we cannot keep stuffing the curriculum with everything.

There's a finite limit to what you can put in and what you can expect your students to understand. So, as far as the faculty is concerned, they have to have that ability to get rid of stuff, which may not be relevant anymore, or originally have any direct impact on the students learning. And this is true of many things. We had a lot of experience in turning to models to do in anatomy for example, we no longer input, very expensive cadavers costing 10,000 dollars per cadaver for the students who come and dissect them. It's no longer necessary unless the students is a surgery student and has to have that skill of dissection. The first-year anatomy is completely changed.

I mean, everywhere you no longer expect students to start from the head and go down to the toe dissecting every bit and piece nerve and muscle and so on. So, I think this is also an instructive way of doing a correction in terms of the curriculum itself, what we need to put in and what is dispensable. We can safely say that this is no longer relevant to the product that we are looking to produce a doctor who is not going to be a surgeon, but is going to be a generalist, so to speak that he's a safe doctor. So, we need to also take this opportunity of online instruction to keep monitoring the content of what is being delivered. So, I mean, my colleagues also have had direct experience in this maybe we need to do hear from their own experience what's been like in teaching microbiology online.

Dr. Archana:

Thank you. Thank you so much. Anyone else would like it?

Prof. Shahid:

Dr. Archana, so, if you allow me. So, I have this chance to take, you know, practical sessions with the students. So, well, I mean, you asked about online teaching. So, if you look into the microbiology stuff, you know, that we deliver in our problem-based curriculum. So, we have to realize that here the microbiology that we give to students is not very in-depth microbiology. So, because traditional microbiology is something very different and in problem-based learning here, the microbiologist stuff, although the concepts are covered, but not so in-depth like we deliver for our traditional teaching. Well, so the labs basically I mean what I have seen. There are two types of labs, so one is more or less like theoretical based and we call that as dry labs. And the second is supposed to be a kind of wet lab

where we have to demonstrate some experimentation done in front of students or if there are any more volunteers from the student side, they do themselves.

So, well, my experience for online is as far as dry laps are concerned. So, these online sessions are pretty good. They're very good. We can show them by delivering the PowerPoint and we can stop and discuss on those parts. We can highlight on in online mode. And even in online, we can show them videos of whatever we are talking of. So, this way it is very good. But here we have to realize that if there is a wet lab, so that cognitive skills, basically they are missing out the students altogether.

So, if you see in microbiology, the microscope is the major, the most important basic equipment and the students if they are attending purely online, they have not even touched the microphone. So, the focusing of a slide under the microscope another basic step in microbiology is for example, gram staining is very common in microbiology for identification for every organism, at least a gram staining is done for bacteria, right. So, they don't know how to stain, how to focus, how to handle a microscope. So, these are concern. I mean, we can't show them in the videos, like, say this is slide, put it under microscope and focus it. When it comes for course adjustment and file adjustment these skills are really missing. We can show them photograph on (00:20:00) online mode. So, my concern basically is missing out of cognitive skills.

Male Speaker 1:
Can I interrupt?

Prof. Shahid:
Yeah.

Male Speaker 1:
I mean, to be the devil's advocate, I mean, somebody could turn on and say, what's the point of a medical student learning how to stain a slide and look down a microscope. He just takes a sample from the patient and send it to the lab.

Prof. Shahid:
I can answer that Prof, you know, internal medicine and emergency medicine and all those, emergency, the physicians, basically they have a side room anywhere. So, they can take

directly the specimen, for example, CSF tap, they have a side room. They have to look under the microscope to start empirical therapy because delay in some sample might create problem. You may have false negative kind of results. So, if any emergency medicine physician they should know how to work in the side room at the bedside. So, they have to take the sample and they have to observe under the microscope by themselves and another sample, they have to send to lab for confirmation. So, every physician should know how to handle a microscope, how to make a slide, how to observe under the microscope, if not the stained one, at least the wet one.

Male Speaker 1:

I think that's a very important issue because we sort of think that every student is going to end up in a tertiary care hospital with all the facilities and all that. I mean, many of these students going to be working in rural practices or perhaps sent to remote places and they are on their own more or less and many, many of these. And so, they need to have these basic skills that you described of a side room lab, which the doctor can make a diagnosis within five or 10 minutes and decide on the course of management. Yeah.

Prof. Shahid:

So, my opinion was like so online is good for theoretical part, the dry labs and where there is something like to be given as a cognitive skill. So basically, we are missing out that and it can be only given as a face-to-face. So, my input is like a blend of online and face to face, in my opinion. For practical microbiology is going to be a good option rather than going entirely on the online mode or entirely on the face-to-face. So, this is my suggestion actually my view.

Dr. Archana:

Thank you, Professor Shahid. I think Dr. Eman has a class at 10:30. Has she left? Okay, no problem. Anyone else want to share your experience or views about? Dr. Eman is back. Okay. Dr. Eman, can you hear me?

Dr. Eman:

Yes, I can.

Dr. Archana:

You can proceed because I heard you have a class at 10 30. You want to leave early. You can share your thoughts. Yes.

Dr. Eman:

Okay, my concern, I am involved in the immunology curriculum. So, we have some labs that are dealing with immunology. Regarding the online and the current situation, most of the points that are mentioned by Dr. Shahid apply to our discipline mainly the subspecialty immunology where dry labs are easily given and the message is given to the students that we want to pass. And we are doing like videos in order to be alternative to whatever skills or laboratory methods that we want to explain. The deficient point as mentioned by my colleagues is the feedback from the students and what skills did they take and to what extent they understand and grasp this interaction really is missing. Although, we always have the open questions and so on, but still as a skill, we cannot say no evaluate or see. But as a teacher, from our side, I think most of the main message that we would like them to see in the laboratory immunology part are actually passed and given by alternative ways, as I just mentioned.

Dr. Archana:

Thank you, Dr. Eman. Anyone else want to contribute for the first question, which I asked your experience on online microbiology lab session.

Prof. Shahid:

If you allow me, I can request anyone from if we can hear from Ali. Ali, can you share your experience please?

Ali:

Yes, Prof. Sorry, (00:25:00) actually, I was just listening because I'm trying to put things on my discipline. The thing is about we are talking about the MDs, I mean the medical students. What about the MLM sessions? Are we in a position to talk about?

Prof. Shahid:

I think this is basically for MB. Dr. Archana, can we cover MLM here or only the MDs here like, you know, because medical students we are talking about?

Dr. Archana:

Yes, mainly for undergraduate curriculum we are talking about, yes, yeah.

Ali:

Okay, I mean, no, actually I was just having some concern about the people who are continuing in their career. So, what is, I mean, what will be the output later on? So really, I don't have much to say because all covered by Professor Mohammed and Dr. Eman also. So, I don't have much to say. Yeah.

Dr. Archana:

Thank you. Our colleagues brought out some of the advantages of online teaching already in the previous discussion. Any more advantages you want to add for online microbiology lab session? You think online has any advantage apart from what has been mentioned, would you like to add something?

Female Speaker 2:

Dr. Archana?

Dr. Archana:

Yes.

Ronnie:

So, when we compare face-to-face like in face-to-face, we have groups, groups of students, they come and they attend the sessions. But in online, we could accommodate all the students at a time and they could go through the slides because when it comes to face-to-face, some of them, they sit in front so they can see the slides and those are behind it's difficult for them to understand as well as see this, especially when it comes to a case discussion and all. So, like when you see that online sessions are good, especially if they are dry labs, as Professor Shahid said, because we can accommodate all the students and they can go through the slides and the talk, because every time you repeat, maybe you miss something, but here all these students attend a single session and the information is complete to all the students. So, like that, I think online is better for dry labs. Like we can cover everything at a time.

Prof. Shahid:

I think if I may, again Ronnie makes a very good point, but I would slightly disagree on something in that online discussion between the instructor and the students, generally. In a lab, you quite rightly say that much of learning is also done in groups of students meeting together that are signed up for specific task or the rotate or whatever. I think we should not forget the students learning from each other. In a group session, they learn from each other and you also actually somehow indirectly assign a role to a student for leadership. Then you can begin to recognize who's a leader or leading a student in terms of instruction, in terms of helping them tutor or the teacher to increase the capacity of the whole class in terms of the process. So, I think these groups within the class, which is not really possible in an online session where there's just one mass of students who are sitting quite often they might switch off or even everything is switched off and the mind is switched off as well, because they are not there. But when they are inside in front of you, they cannot switch off really. So, whether they are altogether or in groups, I think that part of learning group learning is also an important contribution to the process of acquiring skills, as well as knowledge.

Dr. Archana:
Thank you. Anyone else who wants to add?

Prof. Shahid:
I agree with professor, I'm sorry, Dr. Abdur Rahman go ahead please.

Dr. Abdur Rahman:
Good morning, everybody. Thank you very much for this meeting. Sorry, I was in the car and I heard whatever said. Yes, I believe this kind of compromise, as we know all because of this Covid from my experience you will deliver whatever you will deliver in the lab. We substitute with the small video clips for the thing which was practical, like let's say gram staining. You will do a small video clip and you will put that for the students. The good thing is this thing is recorded and the student they can see it any time they want. This is one good thing. The bad thing that the attendance is very poor around maybe you don't know, 50% or less. And from this 50%, you don't know how many of them (00:30:00) they are engaged with you. So, this is the only negative thing about it. And when you ask a question, very few students will answer, usually very few. I don't know, 1, 2, 3, 4 students will answer your question. So, the only thing which I want to add in addition, I am agreeing whatever our colleagues said about. Thank you.

Dr. Archana:

Thank you. Yes, Professor Shahid you wanted to say something.

Prof. Shahid:

One of the points Dr. Abdur Rahman has already mentioned that in online we record the session so students can revisit it later on whenever they wish to. So, this is another important aspect of having online sessions, especially for the dry lab. So, I'm favoring online for dry labs only. Well, I agree with Professor about the attendance, so we don't know how many of them are attending online, but on the other hand, I would also like to say that, you know, in face-to-face attendance is not a requirement for our students. So, it's up to them. So, in some of the sessions, you will only see like, you know, 20 students visiting 25. So there is no such a difference between online attendance and the face-to-face attendance, unless we make it compulsory for face-to-face. ___.

Male Speaker 1:

Dr. Shahid it's not a matter of how many are there, whether it's one or a hundred, it's a measure of engagement whether they are able to interact, not only with the instructor, but each other as well. So, if they're even a few students present, but they are talking to each other discussing or helping each other, that's a process of extremely important in terms of, you know, experience of learning altogether. So, I think it's not simply making it something compulsory. I mean, I agree. To my mind, I don't think they should be compulsory that they must attend everything. It's their choice. In fact, if they don't like something, they walk away, they actually vote with their feet, you know and that's also a reflection of the students' perception of learning something from that session. If they're regularly not attending, that means there's something wrong with the session. The instructor is very dull or they can't find anywhere else, particularly in dry session. I mean, there's some very good packages available now on various sites, they can look up at their own leisure. So, why should they attend? So, I think we need to also look at this business of making students attend things compulsorily whether it's a lecture session or a lab session, whatever. What should we made clear from the outset is it is for their benefit and I agree with that perception is right up to them.

Prof. Shahid:

I do agree Professor, you know, that's why I mentioned like there should be face to face lab but not for the dry labs for wet labs, not for dry labs, because, I mean, there is no point as Ronnie has raised a very important point. Either you have to repeat it four times for the entire group, or you have to call all of them in a single room and to deliver whatever dry level stuff is to be given. So, I'm just in favor of that part. And second thing that, that the group discussions or making a small group. So, there are ways out in online teaching as well. We can make a small room.

Male Speaker 1:
Yeah, break out rooms and so on, but that doesn't always work. Come on, give me a break.

Prof. Shahid:
Alright. Okay.

Dr. Archana:
Okay. Now thank you for all your input. Now, let us move on to the next question. Some of the disadvantages of online teaching has already been mentioned. Would you like to add something to disadvantages of online lab session, anything more apart from what has been said?

Prof. Shahid:
One disadvantage, you know, that's what I'm thinking, you know, it's coming in my mind, like, you know, suppose this year I have delivered any session, practical session online it's already recorded. It's only there with the students circulating with them. So, next time, if I'm going to come again and giving them online, so probably they will not attend, they already have it. So, this is one of the disadvantages I would say, like, you know, so they have all the things well in hand.

Dr. Archana:
Thank you. We have Dr. Khalid ___ with us. Okay.

Dr. Khalid:
Yes. Good morning.

Dr. Archana:

Good morning. Good morning. Thank you for joining us. Now we will go to the second component.

Dr. Khalid:

I have been listening.

Dr. Archana:

Dr. Khalid, (00:35:00) would you like to contribute anything on online session? Now, we are moving towards a face-to-face. Would you like to contribute something on disadvantage or advantages?

Dr. Khalid:

Okay, you know, yeah. I mean, let's, let's look at actually what we've been doing in the lab, in the physical lab because of the large number of students and you know, the lack of enough supervision and materials and so on the labs basically are show and tell. The students gather in groups of maybe 20 around one station, and somebody has, and they have material on display on the bench. And there is somebody there either one of our technicians or faculty members explaining to them, you know, what is this and what happens and so on. And in the best of situations, one or two students are actually asked to do something while the other 18 are watching. Now in the online, the same thing happens, but it is videotaped and played back, so the show I'll tell is in video, instead of in live, there is no practical either way. Maybe two students, but I mean that doesn't affect the other students in any way to acquire any skills. So, the show and tell is either done physically or done online. Now, obviously physically you can see the students with you and maybe there is more of a pressure to be there. In the online, you can just sign in and that's it and not listen or not here. Nobody sees you and you don't participate.

So, there is maybe a lesser chance of actually listening in or watching this show and tell. But as Professor Shahid said it is already there on tape. They can play it anytime and watch it. Isn't that part of self-learning being able to do things at their own pace whenever they want, rather than be forced in time and place. Yeah, now obviously the other thing about the live lab is that when they see the material that is being displayed, there is a better perspective both 3D, you see it in 3D rather than 2D as you see it in the video. And you are able to comprehend a little more about the size of things, rather than just, you know, you don't know how big that thing that you see there until somebody puts maybe their hand on

the door or whatever. So, yes, there is a great advantage to seeing it live than online, but again, it's a single time. If you don't come to the lab, you lose it. If it is online, you can watch it any time. So, there is some advantage here and disadvantage. Skills I think, the amount of skills that we teach them is really limited. There are some basic things that we feel they have to experience let's say maybe not become a proficient at doing these skills but at least experience them.

So that at least they appreciate, I think they appreciate the work that is done by the lab. And also, I think it also gives them a little better understanding of the (00:40:00) reliability of the results let's say. And the difficulty sometimes of getting a definite result when they see it, when they understand, you know, the steps that are involved. So perhaps when they get a result, which is unexpected when they are managing a patient, they might weigh it and decide to ask for a retest or maybe lessen the dependence on the test and more on the clinical signs and symptoms, you understand. So, they appreciate the technique and understand its limitations. I think that is really the thing, very few doctors at this time, actually do any testing themselves. It all comes from a lab. That is very, I mean, I don't see it emergency, they say emergency, or maybe if you are out in the woods or something, you know, I don't know whether you have the equipment to do the test anyway. So, there are pluses and minuses for both systems, but definitely the human interaction of the live session I think has a little more advantage. I would like to see that, but I would also like to see that perhaps there is a video of that so students who are not able to go or students who want to see it a second time are able to see it.

Male Speaker 1:

I think if I may Dr. Archana just to push Khalid's points a little bit further and that is in the overall training of our medical students. I think this element of curiosity that the student gets or to experience in a live session with this instructor should be provide a sort of an impetus for some of our medical graduates, not to end up prescribing pills and putting injections but towards research, towards finding out things, why things happen the way they happen. I think this is for a lab science, like microbiology, it's a good introduction towards research and at this level of undergraduate training, if they get interested in that, they can follow up their later professional lives a better understanding and a better sort of a direction for their career development.

Dr. Archana:

Thank you. Thank you. Now we move towards the face-to-face learning. You have taught both online and face-to-face. So, you have experience of both. How you felt, what was your personal experience or what was your perception when you taught sessions in campus, face-to-face when you saw the students in-person how you felt, what was the feeling? What was the experience you felt?

Male Speaker 1:
Well, there's no comparison. I think as I'm concerned as a face-to-face teaching, there's nothing to beat face-to-face interaction because to me, this process is not simply me delivering something and they're sitting there receiving something it's always and spontaneous. You know, when you're doing it online, there is some sort of hesitancy, quite a lot of it hiding behind the anonymity provided by the medium. And there isn't the same amount or quality of interaction and that is to me, the main thing that's missing in the online session.

Dr. Archana:
Okay. Thank you. Would you like to add any more advantage for face-to-face teaching? Any other advantage apart from what has been discussed? Anything else you want to add?

Female Speaker 2:
Yes, the face-to-face you cannot compare it. Face to face you are feeling the student with you. A lot of time, we see their emotions and sometimes we know the student that he has a problem. Let's say he is not happy. He is sad. He is whatever. Then we can detect all of this in face to face as some doctors report to us on student affairs that we have such student, we feel he is sad. He is having problems in the family, another student he is having like (00:45:00) some kind of whatever psychological problem. So that element is missing.

Dr. Archana:
Okay. Thank you. Now, let us go to the next question. What are the disadvantages of face-to-face session? What is the other flip side of face-to-face session in microbiology lab?

Male Speaker 2:
But as we said, when we did these face-to-face because of the limitations I always wished we could do proper labs like the ones we had when we were in college that is having each two students with their own microscope and bench and materials and instructions and let

them do it themselves and then have the supervisor circulate and watch. But you know, the limitation of the material and the supervision and the large number of students and so on was a little prohibitive. So, we ended up doing these demonstrations show and tell sessions. Now the disadvantage there is if you have 20 students in what we are calling a station and they are trying all to see and listen to what you are trying to say, because of the large number, there are some people who don't see very well from the angle they're in, in the second row, cannot hear very well because you are facing another direction.

So, there is some disadvantage there because of the larger number and the inability too. Also, there is a limited time because what we've had to do was take the 200 students and make them into two groups of 100 and work in two labs. So, 50 students in each lab and we have only two hours. So, one hour for each group, right, for each group of 100. And if you have two stations or three stations, you are talking about half an hour or 20 minutes for each station, 25 students maybe in a group, larger numbers and difficulty. So, there is some limitation there I see with the face. When you tape it, at least it is like you and the professor alone, you can see everything, you can hear everything. So, there is an advantage there and actually in some sessions, we've also included video in the session. Pre-taped stuff that we show them during the session so they can see something maybe that we cannot demonstrate.

Dr. Archana:
Thank you. Yes, Dr. Ali.

Dr. Ali:
If I have to add here, there is one point that I noticed while teaching the practical sessions for the face-to-face and it is related to the interest of the students. The students from one level to another, if you look at them from year one, now I'm teaching even year one practical sessions biology so from there you're going up, year two year three, they lose the interest of any practical handles to do. And you'll see the group of the students not all of them are interested when you take a group of 15 or something. They're not all interested. They just came to see for the exam. What is there in the exam? And they are not interested to do a practical maybe because we lost the observation stations in the exam that's why they are not interested. And they felt they can answer everything, even if we have the spot exams. That's why, I mean, we have this problem of interest. When you ask the student to

do something, as in hand, he will not come. You cannot force him to do it. That's what I noticed during the practical session.

Dr. Archana:

Thank you very much.

Prof. Shahid:

Yes. Here, what I'm realizing we are not debating, you know, the online versus face to face as a global kind of right. We are discussing what we were delivering face-to-face in our curriculum in CMMS, department of microbiology and what we did this year in the last year, (00:50:00) in the Covid era, like online teaching. So, it's not a universal discussion between whether this is better or that is better. I mean, my perception I'm taking this forum discussion in a way, like, you know, comparing our own stuff that we were delivering before face-to face in department of micro practical sessions and online in micro. So, Dr. Tabbara has raised a very important point here. We have to really see how we were delivering our microbiology lab face-to-face okay and the other point Dr. Ronnie has mentioned, and that is very valid again. So, you have to repeat four times the same stuff to the students and you might miss out some points in the second group or third group or fourth group. So, you might have given this stuff, this important point to one group and you have missed out with other, and you have raised some important point with other group. Ali has also raised that important point. It depends on the interest of the students.

Probably, I mean, they don't have, when you repeat the same thing and if you see, like, you know, the students are not interested. So, you are not interested in telling them again, you know, so that's what I have seen. First group was enthusiastic. So, I was also, you know, and I delivered many things. Third group was least bothered. Nobody was listening. So, khalibali, I also did the same thing. Okay, so I mean, yeah, if you are not willing to listen, so don't listen, I'm not going to tell you, right. So, if you see the online mode, at least the theoretical part, if it is for the entire group. So, we can at least that stuff we can deliver in entirety, in totality to the entire group so that nobody has missed out. This point was raised by Ronnie and practical. So, my opinion is we have to really look into the ways how we are delivering our microbiology lab. Okay, so we are making four groups, four instructors and the repetition of the stations, the instructors are telling them the theoretical part also, and the practical part in there. So, my opinion and my suggestion for microbiology lab is we have to have a first theoretical part for the entire group in the beginning and then the

practical part right. Now here, the instructors will not tell them anything rather they are as the facilitators. So, the practical parts, hands-on the students, they have to do hands-on or something like that.

So, I'm seeing our microbiology, like it should be, I mean, this is my personal opinion. A blended way of delivering the microbiology lab in our curriculum could be a fruitful rather than, you know, going entirely to face to face or entirely online teaching. So, we have to really look into how we are delivering and what modifications we have to do in our labs. So, wet labs really are missing in microbiology. So, I'm in favor of having more wet labs where the students they do by themselves because Dr. Tabbara has raised another very important point, feal and touch, you know. The students, they will feel, you know, that they will touch the culture media, they will touch the slides. They will feel, they will touch the microscope. So that particular component is really missing in online. I agree with that. So, we have to really modify our system. So, I'm in a balanced way, like online versus so both can go hand in hand. This is my opinion. Yeah.

Dr. Archana:
Thank you, Professor Shahid. Actually, that was about to be my next question. We have seen both the worlds now, both the worlds positive and negative, we have discussed in detail. So, will it not be a better approach do you think it will be a better approach to take the best of both the worlds, combine together and make it as a single package for the students to come? This is what we call it as blended learning, where we have the online component for whatever advantages we have said. We also have face-to-face teaching for whatever advantages we have discussed. Do you think this blended learning mode will be a better option for microbiology lab sessions? What is your opinion do you think?

Prof. Shahid:
My opinion is yes. My opinion is yes

Dr. Archana:
Okay. Thank you. Anyone else has any other opinion?

Dr. Abdur Rahman:
My opinion is yes also because we can shift the theory part of it to online and we keep the hands-on of it, keep it in the lab and we can shorten our lab or we make it available for our

students in smaller groups. Yes, we can do it. It will be a good. In addition, it will be recorded. It would be for the students anytime they can see. We kind of provide short clips, (00:55:00) video films, we produce it ourselves as we've done. So, that is our property and it would be very nice to have this blended time.

Dr. Archana:

Anyone else. Yes, please.

Male Speaker 3:

Yes. I do agree with what Professor Shahid and what Professor Abdur Rahman to have this blending learning the theoretical part which will be taught online. I prefer if we concentrate on the specimen collection and transportations, this is very important objective, which we have to deliver to the students. And also the lab sessions or test which we are going to teach in the actual skills should be taught online. I mean, the principle of the test and the correlation of the test and the actual clinical, for example, when we teach coagulase test, catalyst test. The principle of the test can be taught online but the most important thing is to give the correlation between the test and the actual clinic and I mean in vivo. And then for the skills, I think we have to select some test, some other set that related to the clinic.

For example, when we say the carbohydrates, the media, lactose fermenter or glucose something like that, we have to tell them the need of the organism to the carbohydrates. And when we give the tests, for example, in the CSF, sometimes you find the presence of bacteria is decreased of the glucose. This is a correlation between the clinical and the lab, and also if possible, and I think this needs to work for it. Before in the 80’s, we used to have two types of exams. We used to have lab skills exam plus OSP, before we call it spot exam. Now it is OSP, then if we can go back to have both lab skills exam and OSP, that will encourage the students to attend the lab. And at the same time for the instructors and who are preparing the test, I also encourage them because if the number of the students who attend the lab, there are few and you are preparing so for say 200 students, they come 50. Then it will upset you, this means if we can have lab skill exam and to select some tests, not all of them, some tests, which are related to the clinical to be taught as skills. Thank you.

Dr. Archana:

Thank you. Thank you so much, sir. Point well-taken.

Prof. Shahid:

Dr. Archana, if you allow me.

Dr. Archana:

Yes.

Prof. Shahid:

Ahmed has raised a very important you know, suggestion here. I mean, if I'm not wrong, we discussed it before as well in one of our department discussions, maybe. So, lab skill examination, because we are talking more about face to face, laboratory face to face is better than online part. So, face to face is going to be better if we have real skills development, because this is practical, this is not theoretical. The forum discussion is for practical part, practical microbiology, right. So, if we have those hands-on wet labs, right, so then only we can say like face to face is going to be more important. So, Ahmed has raised a very important point, there should be a lab skill examination, but to organize a lab skill examination is going to be a difficult task. So, that's what I know.

So, what we can do, I mean, we have to really look into our curriculum, the practical curriculum. And we need a big modification there. So, from dry labs, we have to shift towards the wet labs. And then we have to really identify our specific wet labs that these are the specific wet labs and these are a specific wet objective that students they have to perform. And we ask the students to do in the labs and the instructor who is present there, he or she is going to mark them. So, this is going to be a kind of continuous assessment and a specific portion of this continuous assessment, like 10% or 20% should go towards OSP (01:00:00) examination. That's what we are doing like in tutorials, 20% tutorials and remaining is the examination. So, for the practical part, we can have like 10-20% for these skills, continuous assessment lab skills, and remaining can become OSP. And this way, we can even motivate the students who are attending, come in person because they know now 10-20% marks are based on those skills, actually. So, this could be another, I mean, this is very good position and I'm in favor of that. We have to modify our curriculum that way.

Dr. Archana:

Thank you. Thank you so much.

Male Speaker 2:

Also, in the OSP exam, if we introduce the observation stations, this is very important for microbiology as for in the OSP observation.

Dr. Archana:

Thank you. Thank you so much. Now we are going to the last question of today's discussion. In your opinion, how do you think we can deliver lab session addressing the topics like sample handling, processing manual, or machine or automated and interpretation of the results. These are the topics which mode do you think online, face-to-face or blended for sample handling, processing and interpretation of the results. These are the few sessions, which want your opinion.

Prof. Shahid:

Sample processing it is self-explanatory like processing is processing, how the sample is to be processed in the lab. So, it's not going to get theoretical. It has to be done face to face. They have to handle the specimen even if it's assimilated specimen. So, for example, a distilled water can be given as a urine and they should know how to process that specimen. It is going to be face to face. Interpretation of the result, so here in microbiology, we have two types of interpretation, a slight focus under the microscope, but what is the interpretation? Okay, that's going to be face-to-face but interpretation of the report itself, the culture report is this, estimate is this or CSF, you know, the reports are these, so that interpretation can be online. So, it all depends what interpretation we are talking about. Is it like interpretation under the equipment or the microscope or a culture plate? Something like that is face-to-face or printed interpretation, report interpretation can be done by online. It depends.

Female Speaker 3:

Can I add something here?

Dr. Archana:

Yes, please.

Female Speaker 3:

Regarding the interpretation, I think the use of case studies as examples of different scenarios and how the different results can be interpreted. This can be given online and this

can be given like in the mode of discussion of different cases and then trying to teach them how to interpret these results, the lab results in these different cases, clinical cases. So, this objective can be done online by another way, can be.

Dr. Archana:

Thank you.

Female Speaker 3:

Even the sample processing, sample processing also I'm thinking of how can we make use of the online in order to do the objectives that we have? So even sample processing, we can do it also, but what is missing in the online is the interaction which we should think of ways to do it in order to overcome that. Even if we make it in the groups, we can make groups online. We can make a type of smaller groups rather than just all of the group attending and having the interaction between the tutor or teacher and the students and showing them always like videos and the pictures, like, for example, the difference between saliva and sputum and that different types of sample is not the quality of the sample. So, all these interactions is a skill and a message that we want to pass, we can think always of alternative ways to do it online, which is now the method that we have to make use of more, to try to deliver the messages that we want to pass to the students and the skills that we want them to gain. Thank you.

Dr. Archana:

Thank you. Thank you so much. Now we have come to the end of the session. Before we conclude anything else you want to say which is not covered in the previous questions, which I asked you want to say something. (01:05:00) You want to add something to the discussion?

Female Speaker 3:

I think we have learned really with this experience of the Covid pandemic in trying to accommodate and do all what we used to do in traditional methods, by using the online and especially in the lab, it was not easy, but I think that we can modify more and more, and we have gained the experience from that. Like the method, many of the disadvantages mentioned today, we can think of ways to overcome it. Like students are not engaged with you, how to do that. Students are not attending and just showing up signing up, how to do that. Like we are doing in tutorials, in tutorials, we have smaller groups and they can just

show the name that he's there, but the tutor can easily pick up who is with him and who's not. So, I mean, that we have learned and we can modify more and more the online to achieve better results rather than the current experience that we were doing like dry labs and small videos. We can think of more of more ideas in order to improve it and overcome all the disadvantage that are mentioned today.

Dr. Archana:
Thank you. Thank you.

Prof. Shahid:
Sorry, you are done?

Dr. Archana:
We are going to complete, but we have one more minute or two more minutes, doesn't matter.

Dr. Ali:
Okay, it's related to our images and videos, which we have done as our colleagues, you know, here that we have done this on our own effort, but I think we'd still need specialized people to do the videos and images for us because we had that company who helped us in the videos, but now their contact has ended. So, we still need a support on videos because if we want to improve the video, we need a real specialized people with that and even for images. Yeah.

Dr. Archana:
Yes. Thank you. Dr. Tabbara, you wanted to say something?

Dr. Tabbara:
No, not really. I think most of the ideas were covered by everybody. Yes. Thank you.

Dr. Archana:
Thank you. I just want to say a few things before we conclude. It's not only our university globally all universities, all the departments are now reflecting upon their experience and the people are coming out with the new solution for whatever we have discussed. So, hopefully near future, we'll have more options to explore, to overcome the advantage and

disadvantage of both the ends of the spectrum and as pointed out, it's time for us to also have a curriculum review and see what fits us best in our institution for our department. So, hopefully we will also taking this up in future and as Dr. Ali mentioned, the technical support is when we talk about online is we have to think of a core team which can help us in training and also making it more refined for our students. Hopefully, things will be better in future. Thank you very much all of you for your valid input. Let us take it forward. We'll try to make a report and we will submit back to you for your reference. Thank you so much. Thank you.

Dr. Tabbara:
Thank you.

Female Speaker 3:
Thank you all. Thank you.

Male Speaker 1:
Thank you very much.

Dr. Archana:
Thank you.

Male Speaker 1:
Thank you.

Dr. Archana:
Thank you.

Male Speaker 3:
Thank you.

Dr. Archana:
You didn't talk Shahid I mean Shadab.

Male Speaker 4:
Since so many people are talking. I don't think I had something to input.

Dr. Archana:

Really, your input is also valid. No, that's okay, no problem. I am happy you joined us. Very good. Thank you.

Male Speaker 4:

Thank you.

Dr. Archana:

Thank you. Bye.
