# Supplementary material for: Perception of online and face to face microbiology laboratory sessions among medical students and faculty at Arabian Gulf University: a mixed method study
Source: BMC Med Educ. 2022 May 30;22:411. doi: 10.1186/s12909-022-03346-2 (PMC9149330; doi:10.1186/s12909-022-03346-2)
Supplement: Supplementary file 4 — Additional file 4. [file 12909_2022_3346_MOESM4_ESM.pdf]

YEAR 3 YEAR 4 STUDENTS RESPONSES

Timestamp	consent to participate in this survey	Gender	Age in years	Year of study	Amount of time did you spend on each online practical	Where did you work on the online practicals?	The f2f labs were enjoyable	I preferred the f2f labs	f2f labs enhanced my understanding of the course	All labs should be f2f	The online labs were enjoyable	I preferred the online labs	Online labs enhanced my understanding of the course	The different types of interaction (animations etc.) in the online labs improved my learning	I found it difficult to follow the flow and meaning of the subject material in the online labs	I consider the online labs to be an essential addition to traditional f2flabs	Technical enablement (internet/software/hardware) of online sessions was satisfactory	All labs should be online	The best option to deliver microbiology lab sessions would be through one of the follow[illegible]	Give reasons for your response to the above question	Compared to other AGU medical scho[illegible] value to the MD curriculum? Select on a scale of 1 to 5, where 1 - no value, 5 - very high value
2021/05/06 10:49:40 am GMT+3	Yes	Female	21	Year 4	60-90 minutes	Both	SA	SA	SA	SA	N	D	D	SD	SD	SD	SD	SD	FACE TO FCAE sessions only	its more engaging leading to a better understanding	5
2021/05/06 10:50:15 am GMT+3	Yes	Female	23	Year 4	60-90 minutes	Off Campus	N	N	N	D	SA	SA	SA	SA	D	N	SA	SA	COMBINATION of online and face to face sessions	There are things that f2f would better to see .. but online classes makes it much easier to understand and study	5
2021/05/06 10:52:26 am GMT+3	Yes	Male	21	Year 4	60-90 minutes	Off Campus	N	N	N	D	N	N	N	N	N	N	A	D	COMBINATION of online and face to face sessions	-	2
2021/05/06 10:54:22 am GMT+3	Yes	Female	22	Year 4	60-90 minutes	Off Campus	A	A	A	A	D	D	N	N	A	D	N	D	FACE TO FCAE sessions only	For better understanding and communication	4
2021/05/06 10:56:01 am GMT+3	Yes	Female	22	Year 4	30-60 minutes	Off Campus	A	SA	SA	SA	D	D	A	N	A	D	A	SD	FACE TO FCAE sessions only	Because during face to face sessions, students can look at different types of microorganisms, cultures, stains, and etc… trying to study them during f2f classes and this is one way how we manage to stick the information in our brains.	5
2021/05/06 10:56:20 am GMT+3	Yes	Female	21	Year 4	30-60 minutes	Off Campus	N	N	A	N	A	N	A	A	D	A	A	D	COMBINATION of online and face to face sessions	We need to see the slides under the microscope and sometimes some procedures and techniques are very important to be demonstrated face to face, while the scientific material I find the online method enough for that. both methods are fine but personally I prefer to attend the class and see and participate as I find it b[illegible]	3
2021/05/06 10:59:13 am GMT+3	Yes	Female	22	Year 4	60-90 minutes	On Campus	SA	SA	SA	SA	SD	SD	SD	SD	SD	SD	SD	SD	FACE TO FCAE sessions only	There is an experiment I should do it by my self to learn not just see it online	4
2021/05/06 12:08:12 pm GMT+3	Yes	Male	22	Year 3	60-90 minutes	Off Campus	A	N	A	D	A	SA	SA	A	A	A	N	N	COMBINATION of online and face to face sessions	Because some expirement should be done by the students them self	5
2021/05/06 1:21:43 pm GMT+3	Yes	Female	23	Year 3	60-90 minutes	Off Campus	N	N	N	N	A	A	N	N	N	N	N	N	COMBINATION of online and face to face sessions	Some labs do not need to be f2f	4
2021/05/06 7:58:22 pm GMT+3	Yes	Female	23	Year 4	30-60 minutes	On Campus	A	N	N	N	N	N	N	N	N	N	N	N	COMBINATION of online and face to face sessions	to must understand	4
2021/05/10 11:02:26 am GMT+3	Yes	Female	20	Year 3	60-90 minutes	Off Campus	N	A	N	SD	A	A	A	D	D	SA	N	N	COMBINATION of online and face to face sessions	-	5
2021/05/10 11:13:22 am GMT+3	Yes	Female	21	Year 3	60-90 minutes	Off Campus	SA	SA	SA	SA	SD	SD	SD	SD	SA	SD	SA	SD	FACE TO FCAE sessions only	f2f is much better	1
2021/05/10 11:43:37 am GMT+3	Yes	Female	22	Year 3	60-90 minutes	Off Campus	D	A	N	SD	A	A	A	SA	N	SA	D	D	COMBINATION of online and face to face sessions	First because of the situation now, second i think giving the written part and slide on online is better to understand the subject then we come to the lab just to see the practical part	4
2021/05/10 11:47:18 am GMT+3	Yes	Male	21	Year 3	60-90 minutes	Off Campus	SA	SA	SA	N	D	N	A	N	N	SA	A	N	COMBINATION of online and face to face sessions	Need to see to remember thing better and study as online is very good to understand thing after or before the real life will be helpful	4
2021/05/10 11:57:16 am GMT+3	Yes	Male	20	Year 3	60-90 minutes	Off Campus	A	A	A	D	N	A	N	D	N	SA	A	D	COMBINATION of online and face to face sessions	More convenient	4
2021/05/10 12:06:04 pm GMT+3	Yes	Female	21	Year 3	60-90 minutes	Off Campus	A	N	SA	N	SD	SD	D	SD	SA	N	N	SD	FACE TO FCAE sessions only	Difficult to understand the online lab	3
2021/05/10 1:36:03 pm GMT+3	Yes	Female	20	Year 3	60-90 minutes	Off Campus	D	D	D	SA	A	D	N	A	SD	A	N	SD	ONLINE sessions only	no need for face to face session especially when it comes to microbiology. but other subjects face to face may be batter	5
2021/05/10 2:14:14 pm GMT+3	Yes	Female	21	Year 4	30-60 minutes	Off Campus	A	A	A	N	A	A	SA	SA	N	SA	A	N	COMBINATION of online and face to face sessions	Because some labs we have to see the specimens and real picrures but some labs are not	2
2021/05/10 2:59:35 pm GMT+3	Yes	Female	21	Year 4	60-90 minutes	Off Campus	A	A	A	N	N	N	N	N	N	N	N	D	COMBINATION of online and face to face sessions	Material can be available online	4
2021/05/10 3:06:28 pm GMT+3	Yes	Female	21	Year 4	60-90 minutes	Both	SA	A	A	A	SA	SA	SA	SA	SA	SA	A	A	COMBINATION of online and face to face sessions	Some procedures need to be done face to face while the other enough to be given online	4
2021/05/10 4:09:20 pm GMT+3	Yes	Female	22	Year 4	60-90 minutes	Both	N	N	N	SD	SA	SA	SA	SA	N	SA	A	N	COMBINATION of online and face to face sessions	-	4
2021/05/16 11:09:28 am GMT+3	Yes	Female	21	Year 4	60-90 minutes	Off Campus	SA	SA	SA	SA	A	SD	SD	SD	SD	SD	SD	SD	COMBINATION of online and face to face sessions	Sometimes online session are in our hand and can be stored for revision	1
2021/05/18 9:39:49 am GMT+3	Yes	Male	21	Year 3	30-60 minutes	Off Campus	N	N	D	A	N	N	D	N	A	A	D	A	COMBINATION of online and face to face sessions	For more convenient timing	2
2021/05/18 9:47:30 am GMT+3	Yes	Female	23	Year 4	60-90 minutes	Off Campus	N	D	N	SD	A	SA	SA	SA	SD	SA	SA	N	COMBINATION of online and face to face sessions	There are things where its better to see them in nature .. but on the other hand the online classes helped me so much in understanding better	5
2021/05/18 9:47:55 am GMT+3	Yes	Male	21	Year 4	30-60 minutes	Off Campus	A	A	A	A	D	D	SD	D	D	D	D	SD	FACE TO FCAE sessions only	We can interact Easily	3
2021/05/18 9:51:02 am GMT+3	Yes	Male	24	Year 4	60-90 minutes	Off Campus	A	N	N	N	A	SA	N	SA	N	SA	SA	SA	ONLINE sessions only	Because the number of the students might be more than the hall itself and it would be better and easier to be in contact with this huge number online	3
2021/05/18 10:00:35 am GMT+3	Yes	Female	23	Year 4	60-90 minutes	Off Campus	N	D	N	SD	A	SA	SA	SA	SD	SA	SA	N	COMBINATION of online and face to face sessions	There are things where its better to see them in nature .. but on the other hand the online classes helped me so much in understanding better	5
2021/05/18 10:02:16 am GMT+3	Yes	Female	24	Year 3	30-60 minutes	Off Campus	A	A	N	D	D	A	A	A	D	A	A	D	COMBINATION of online and face to face sessions	Some labs are basically lectures on slides there is nothing to experince it by doing to observing, on the other hand some labs has the practical side when we can observe under the microscope for example that needed to be face to face not online and the one that are slude there is no need to face to face interaction	4
2021/05/18 10:07:03 am GMT+3	Yes	Female	21	Year 3	60-90 minutes	Both	A	N	SD	SD	A	SA	SA	SA	SD	SA	SA	SA	COMBINATION of online and face to face sessions	I like when you give us slides of the demo and lab sessions so we donâ€™t miss anything, we can review it after the sessions!.. In addition, it good to be combinat beacause some labs we donâ€™t need to go to the lab to study it, it actually clearer on online class , also i hope you continue to make records of them because it [illegible]	3
2021/05/18 10:11:19 am GMT+3	Yes	Female	22	Year 4	less than 30 minutes	Off Campus	A	A	A	SA	SD	SD	SD	SD	SA	SD	SD	SD	FACE TO FCAE sessions only	Its hard to concentrate in the online classes in general and not anjoyable at all interaction iz zero	5
2021/05/18 10:17:29 am GMT+3	Yes	Female	20	Year 3	30-60 minutes	Off Campus	N	N	A	D	N	N	A	N	D	A	A	D	COMBINATION of online and face to face sessions	Some labs require interaction	4
2021/05/18 10:22:51 am GMT+3	Yes	Male	22	Year 3	30-60 minutes	Off Campus	A	A	A	A	N	N	N	N	N	N	N	N	FACE TO FCAE sessions only	-	3
2021/05/18 10:37:18 am GMT+3	Yes	Female	22	Year 4	30-60 minutes	Off Campus	SA	SA	SA	SA	SD	SD	SD	SD	A	D	D	SD	FACE TO FCAE sessions only	It is a lab I need to see it with my eye and do it with mu own to learn, the online lab will be forgets	5
2021/05/18 10:41:10 am GMT+3	Yes	Male	21	Year 3	30-60 minutes	Off Campus	N	N	D	A	N	N	D	N	A	A	D	A	COMBINATION of online and face to face sessions	For more convenient timing	2
2021/05/18 10:43:33 am GMT+3	Yes	Female	21	Year 3	60-90 minutes	Both	A	N	SD	SD	A	SA	SA	SA	SD	SA	SA	SA	COMBINATION of online and face to face sessions	I like when you give us slides of the demo and lab sessions so we donâ€™t miss anything, we can review it after the sessions!.. In addition, it good to be combinat beacause some labs we donâ€™t need to go to the lab to study it, it actually clearer on online class , also i hope you continue to make records of them because it [illegible]	3
2021/05/18 10:51:46 am GMT+3	Yes	Female	20	Year 3	30-60 minutes	Off Campus	N	N	N	N	D	D	N	N	A	D	N	D	COMBINATION of online and face to face sessions	-	3
2021/05/18 11:18:36 am GMT+3	Yes	Female	23	Year 4	60-90 minutes	Off Campus	A	A	A	N	A	D	N	N	A	D	N	D	COMBINATION of online and face to face sessions	Some subject need f2f stations and it become very difficult online	4
2021/05/18 11:22:07 am GMT+3	Yes	Male	21	Year 3	60-90 minutes	Off Campus	SA	N	N	SA	N	A	SD	D	SD	A	SA	N	FACE TO FCAE sessions only	Cant focus online	5
2021/05/18 11:44:36 am GMT+3	Yes	Female	23	Year 4	60-90 minutes	Off Campus	N	D	A	D	D	D	A	A	D	N	A	SD	COMBINATION of online and face to face sessions	Online sessions are clear but donâ€™t offer opportunity for us to practice.	4
2021/05/18 12:00:30 pm GMT+3	Yes	Male	23	Year 4	60-90 minutes	Both	N	N	N	D	A	A	N	A	D	A	N	N	COMBINATION of online and face to face sessions	Some labs need to be practice by hand and some only by slide online you understand it.	4
2021/05/18 12:21:07 pm GMT+3	Yes	Female	22	Year 3	30-60 minutes	Off Campus	A	SD	D	D	A	A	A	A	D	SA	A	N	COMBINATION of online and face to face sessions	Because of the current pandemic I prefer the online lab	4
2021/05/18 12:23:20 pm GMT+3	Yes	Female	21	Year 3	30-60 minutes	Off Campus	N	N	N	D	A	A	A	A	D	A	A	A	ONLINE sessions only	Much better understood in online	3
2021/05/18 12:27:33 pm GMT+3	Yes	Female	20	Year 3	30-60 minutes	Off Campus	N	N	A	D	N	N	A	N	D	A	A	D	COMBINATION of online and face to face sessions	Some labs require interaction	4
2021/05/18 1:43:04 pm GMT+3	Yes	Male	4	Year 4	30-60 minutes	Off Campus	SA	A	A	D	A	N	A	A	A	A	SA	N	COMBINATION of online and face to face sessions	Both of them are good	5
2021/05/18 3:12:07 pm GMT+3	Yes	Female	21	Year 3	60-90 minutes	Off Campus	A	SA	SA	N	SD	SD	D	N	N	A	N	SD	COMBINATION of online and face to face sessions	F2F labs are more enjoyable and well understood, although not all labs need to be f2f to have the full understanding.	4
2021/05/18 3:17:19 pm GMT+3	Yes	Female	21	Year 3	30-60 minutes	Both	A	D	D	D	A	A	A	A	D	SA	A	N	COMBINATION of online and face to face sessions	Some labs donâ€™t really need attendance since they only explain the slides.	4
2021/05/18 3:50:55 pm GMT+3	Yes	Female	21	Year 3	30-60 minutes	On Campus	SA	SA	SA	SA	SA	SA	SA	SA	SA	SA	SA	SA	COMBINATION of online and face to face sessions	I think itâ€™s better to combine	4
2021/05/18 4:10:22 pm GMT+3	Yes	Female	23	Year 4	30-60 minutes	Off Campus	SA	SA	SA	N	D	SD	D	N	N	D	D	SD	COMBINATION of online and face to face sessions	Presenting doctors slides and materials they want to deliver could be through online sessions. But seeing the tests, microscopes, samples should be face to face.	5
2021/05/18 4:53:01 pm GMT+3	Yes	Female	20	Year 3	30-60 minutes	Off Campus	N	N	A	D	N	N	A	N	D	A	A	D	COMBINATION of online and face to face sessions	Some labs require interaction	4
2021/05/19 12:40:23 am GMT+3	Yes	Female	22	Year 4	60-90 minutes	Off Campus	D	D	N	SD	N	SD	N	A	N	D	A	SD	FACE TO FCAE sessions only	Face to face sessions are enhancing us as students to learn better and to master skills by performing some of tests.	5
2021/05/19 7:20:21 am GMT+3	Yes	Male	23	Year 4	60-90 minutes	Both	N	N	N	D	A	A	N	A	D	A	N	N	COMBINATION of online and face to face sessions	Some labs need to be practice by hand and some only by slide online you understand it.	4
2021/05/19 2:55:38 pm GMT+3	Yes	Female	20	Year 3	30-60 minutes	Off Campus	N	N	A	D	N	N	A	N	D	A	A	D	COMBINATION of online and face to face sessions	Some labs require interaction	4
2021/05/23 9:34:15 am GMT+3	Yes	Female	23	Year 4	30-60 minutes	Both	A	A	A	N	A	N	A	A	D	N	A	N	COMBINATION of online and face to face sessions	Combination , Practical sessions provide us a real views to see materials and methods, even if you do not give us a chance to do the steps of most labsðŸ™‚ðŸ™‚	4
2021/05/23 10:22:30 am GMT+3	Yes	Female	23	Year 4	60-90 minutes	Off Campus	N	N	N	N	N	N	N	N	N	N	N	N	COMBINATION of online and face to face sessions	-	5
2021/05/23 10:27:53 am GMT+3	Yes	Female	21	Year 4	60-90 minutes	Off Campus	SD	SA	A	SA	SD	SD	A	SD	N	SD	N	SD	FACE TO FCAE sessions only	Doing experiments bu hand makes understanding so much easier and we need to learn how to deal with microscopes	3
2021/05/23 10:38:03 am GMT+3	Yes	Female	21	Year 3	30-60 minutes	Off Campus	SD	SD	SD	SD	N	N	N	N	N	N	N	N	FACE TO FCAE sessions only	face to face increases understanding thru practical work	5
2021/05/23 10:43:40 am GMT+3	Yes	Female	20	Year 3	30-60 minutes	Off Campus	SA	SA	SA	SA	SD	SD	SD	SD	SA	SD	D	SD	FACE TO FCAE sessions only	It is more beneficial for us as medical students to attend classes face to face	3
2021/05/23 10:44:05 am GMT+3	Yes	Female	21	Year 3	60-90 minutes	Both	N	N	N	N	A	A	A	A	D	SA	SA	SA	ONLINE sessions only	Micro is a subject that contain a-lot of memorizing so listening to it more than once is very beneficial	4
2021/05/23 10:53:39 am GMT+3	Yes	Female	21	Year 3	60-90 minutes	Off Campus	A	SA	SA	SA	SD	SD	SD	SD	SA	SD	SD	SD	FACE TO FCAE sessions only	Better understanding and more focus	5
2021/05/23 11:17:53 am GMT+3	Yes	Female	20	Year 3	60-90 minutes	Off Campus	N	D	D	D	SD	SD	SD	D	N	N	N	SD	COMBINATION of online and face to face sessions	Face to face interaction gives better interaction.	1
2021/05/23 11:22:59 am GMT+3	Yes	Female	23	Year 4	60-90 minutes	Off Campus	SA	SA	SA	SA	SD	N	N	SD	A	N	A	SD	FACE TO FCAE sessions only	Get better knowledge	5
2021/05/23 12:23:48 pm GMT+3	Yes	Male	20	Year 3	30-60 minutes	Off Campus	A	N	N	N	N	A	D	N	D	A	A	D	COMBINATION of online and face to face sessions	Flexibility	3
2021/05/23 12:46:34 pm GMT+3	Yes	Male	21	Year 3	60-90 minutes	Off Campus	A	A	A	N	A	D	N	N	D	A	A	N	COMBINATION of online and face to face sessions	Sometimes we need to do the test to understand it well	4
2021/05/23 2:05:05 pm GMT+3	Yes	Female	21	Year 3	30-60 minutes	Off Campus	SD	SD	SD	SD	N	SA	A	D	SD	SA	N	SA	COMBINATION of online and face to face sessions	The f2f labs where crowded and it was difficult to see and follow	1
2021/05/23 2:19:05 pm GMT+3	Yes	Male	20	Year 3	30-60 minutes	Off Campus	A	N	N	N	N	A	D	N	D	A	A	D	COMBINATION of online and face to face sessions	Flexibility	3
2021/05/23 3:50:03 pm GMT+3	Yes	Female	21	Year 3	60-90 minutes	Off Campus	SA	D	N	D	SA	SA	A	SA	D	SA	N	N	COMBINATION of online and face to face sessions	-	4
2021/05/24 1:33:49 am GMT+3	Yes	Female	21	Year 3	60-90 minutes	Off Campus	A	SA	SA	SA	SD	SD	SD	SD	SA	SD	SD	SD	FACE TO FCAE sessions only	Better understanding and more focus	5
2021/05/24 4:36:05 pm GMT+3	Yes	Female	21	Year 3	30-60 minutes	Off Campus	SD	SD	SD	SD	N	N	N	N	N	N	N	N	FACE TO FCAE sessions only	face to face increases understanding thru practical work	5
2021/05/25 12:17:27 am GMT+3	Yes	Female	21	Year 3	60-90 minutes	Off Campus	N	N	N	N	N	N	D	N	N	D	N	N	ONLINE sessions only	Better	5
2021/05/25 11:20:54 pm GMT+3	Yes	Male	21	Year 4	30-60 minutes	Off Campus	A	A	A	A	D	D	SD	D	D	D	D	SD	FACE TO FCAE sessions only	We can interact Easily	3
2021/05/26 11:48:19 am GMT+3	Yes	Female	22	Year 4	60-90 minutes	Off Campus	SA	N	A	D	A	N	A	A	D	A	A	A	COMBINATION of online and face to face sessions	Some labs are best delivered online whereas others in person	5
2021/05/26 3:46:07 pm GMT+3	Yes	Female	20	Year 3	60-90 minutes	Off Campus	D	N	SD	N	D	N	A	N	N	D	A	D	FACE TO FCAE sessions only	Able to focus	3
2021/05/26 3:47:51 pm GMT+3	Yes	Female	21	Year 4	60-90 minutes	Off Campus	SA	SA	A	A	N	SD	N	A	D	A	A	SD	COMBINATION of online and face to face sessions	Incorporating online session is an excellent way to enhance our education in situations or lesson plans where an offline class isnt mandatory	4
2021/05/27 1:54:11 am GMT+3	Yes	Female	22	Year 4	60-90 minutes	Off Campus	SA	N	A	D	A	N	A	A	D	A	A	A	COMBINATION of online and face to face sessions	Some labs are best delivered online whereas others in person	5
2021/05/27 2:44:25 pm GMT+3	Yes	Male	20	Year 3	60-90 minutes	Both	SA	SA	SA	SA	SD	SD	SD	SD	SD	SD	SD	SD	FACE TO FCAE sessions only	Online labs are not good for medical student, we need it face to face to interact with each othe and get the information in easy way and to avoid technical problems	3
2021/05/31 8:48:01 am GMT+3	Yes	Male	21	Year 4	60-90 minutes	Off Campus	SA	A	SA	N	SA	A	A	A	N	SA	SA	N	COMBINATION of online and face to face sessions	Some labs needs skill development and some labs need only discussion	4
2021/06/05 5:56:38 pm GMT+3	Yes	Female	22	Year 4	60-90 minutes	Off Campus	A	N	N	D	N	N	N	N	N	N	N	D	COMBINATION of online and face to face sessions	There are some lab need practice	4
2021/06/05 5:56:51 pm GMT+3	Yes	Female	23	Year 4	60-90 minutes	Off Campus	N	A	A	SD	A	N	A	A	A	SA	A	D	COMBINATION of online and face to face sessions	Sessions need to learn it in the labs while others not	5
2021/06/05 5:56:55 pm GMT+3	Yes	Female	23	Year 4	30-60 minutes	Off Campus	SD	SD	N	SD	A	SA	N	A	SD	SA	A	SA	ONLINE sessions only	Online sessions save studentâ€™s time a lot!!!!!!! I recommended using online sessions even after COVID-19.	4
2021/06/05 5:57:52 pm GMT+3	Yes	Male	23	Year 4	60-90 minutes	Off Campus	N	N	A	A	SA	SA	SA	SA	D	A	A	SA	ONLINE sessions only	Easy to back to the information at any time… not like before if you miss the attending..the information is gone and can not be reclamed	4
2021/06/05 5:58:37 pm GMT+3	Yes	Male	23	Year 4	less than 30 minutes	Off Campus	SA	SA	SA	SA	SD	SD	SD	SD	D	SD	D	SD	FACE TO FCAE sessions only	Seeing the lab equipment in front of you and seeing the tests or any procedure in front of you really helps to understand the material well. Moreover, participting in these tests and doing them yourself increases your experience which helps to learn much more effectively.	5
2021/06/05 6:01:56 pm GMT+3	Yes	Male	24	Year 4	60-90 minutes	Both	SA	SA	SA	SA	SA	SA	SA	SA	SD	SA	SA	SA	COMBINATION of online and face to face sessions	Donâ€™t have	5
2021/06/05 6:05:36 pm GMT+3	Yes	Male	21	Year 4	30-60 minutes	Off Campus	N	N	A	SA	N	A	N	A	N	A	A	A	FACE TO FCAE sessions only	Face to face gives you more explanation and makes you understand the problem more	3
2021/06/05 6:20:43 pm GMT+3	No																				
2021/06/05 6:21:33 pm GMT+3	Yes	Female	22	Year 4	60-90 minutes	Off Campus	SD	SD	SD	SD	SA	SA	SA	SA	SD	SA	SA	SA	ONLINE sessions only	more convenient and a lot of time to process all the information from the comfort of home	3
2021/06/05 6:32:15 pm GMT+3	Yes	Female	21	Year 4	30-60 minutes	Off Campus	SA	SA	SA	SA	D	SD	SD	SD	A	D	N	SD	COMBINATION of online and face to face sessions	Discpline is achieved through face to face interactions	4
2021/06/05 6:47:50 pm GMT+3	Yes	Female	22	Year 4	30-60 minutes	Both	SA	SA	SA	SA	D	D	N	SD	N	A	A	SD	COMBINATION of online and face to face sessions	Online for teaching the methods, and f2f to actually learn how to prepare smears and etc	4
2021/06/05 7:11:05 pm GMT+3	Yes	Female	22	Year 4	30-60 minutes	Both	SA	SA	SA	SA	D	D	N	SD	N	A	A	SD	COMBINATION of online and face to face sessions	Online for teaching the methods, and f2f to actually learn how to prepare smears and etc	4
2021/06/05 8:15:26 pm GMT+3	Yes	Female	21	Year 3	60-90 minutes	Off Campus	SA	SA	SA	N	D	D	D	D	N	A	A	N	COMBINATION of online and face to face sessions	Some labs doesnt is theoretical and doesnt need practical applying	4
2021/06/05 10:24:01 pm GMT+3	Yes	Male	21	Year 4	less than 30 minutes	Off Campus	SA	SA	SA	SA	D	D	N	A	N	N	N	SD	COMBINATION of online and face to face sessions	Face to face will be enhanced by online additional sessions	4
2021/06/05 10:51:22 pm GMT+3	Yes	Female	21	Year 3	less than 30 minutes	Both	SA	SA	SA	SA	SD	SD	SD	SD	SA	SD	SA	SD	FACE TO FCAE sessions only	Online not good we are going to be a doctors not computers	3
2021/06/05 11:33:41 pm GMT+3	Yes	Female	21	Year 3	less than 30 minutes	Both	SA	SA	SA	SA	SD	SD	SD	SD	SA	SD	SA	SD	FACE TO FCAE sessions only	Online not good we are going to be a doctors not computers	3
2021/06/06 12:07:39 am GMT+3	Yes	Female	20	Year 3	60-90 minutes	Both	SA	SA	N	N	SA	SA	N	N	SD	N	A	N	COMBINATION of online and face to face sessions	Better	4
2021/06/06 12:08:25 am GMT+3	Yes	Female	22	Year 3	60-90 minutes	Off Campus	A	A	A	SA	N	D	N	D	N	A	A	D	COMBINATION of online and face to face sessions	if the lap was more theory I prefer it online but if there was a part practical it should be better face to face	5
2021/06/06 12:11:39 am GMT+3	Yes	Female	20	Year 3	60-90 minutes	Both	SA	SA	N	N	SA	SA	N	N	SD	N	A	N	COMBINATION of online and face to face sessions	Better	4
2021/06/06 5:35:19 am GMT+3	Yes	Female	21	Year 4	60-90 minutes	Off Campus	SD	SD	SD	SD	SD	SD	SD	SD	D	SD	SD	SD	FACE TO FCAE sessions only	I cannot focus during online lab sessions, face to face sessions help me focus and understand better	5
2021/06/06 10:52:36 am GMT+3	Yes	Female	22	Year 4	60-90 minutes	Off Campus	A	A	A	N	N	D	N	N	N	SA	N	SD	COMBINATION of online and face to face sessions	Sometimes we need to see the slides under the microscope, and other things we need to see it by ourself, thatâ€™s why the online classes are important, but I believe that the online sessions can be beneficial for the solid material, for example we can take the material by an online session and the next day we attend the l[illegible]	3
2021/06/06 10:53:08 am GMT+3	Yes	Female	22	Year 4	60-90 minutes	Off Campus	D	N	D	N	D	D	SD	N	N	D	D	D	COMBINATION of online and face to face sessions	To understand	3
2021/06/06 10:53:26 am GMT+3	Yes	Female	23	Year 4	30-60 minutes	Both	N	A	A	N	D	D	D	N	D	N	N	D	COMBINATION of online and face to face sessions	to avjlid abr diseases	5
2021/06/06 10:55:04 am GMT+3	Yes	Female	23	Year 3	60-90 minutes	Off Campus	D	SD	N	N	A	A	SA	SA	N	SA	A	N	COMBINATION of online and face to face sessions	Because of the large numbers in f2f labs , it is not possible to understand the information well, and we are not involved in the f2f labs much… but it is still interesting to see the process f2f labs	5
2021/06/06 10:55:31 am GMT+3	Yes	Female	26	Year 4	30-60 minutes	Off Campus	N	A	A	A	N	A	A	A	D	A	A	A	COMBINATION of online and face to face sessions	Depends of the material, so if itâ€™s a lecture so I prefer online teaching, if its practical I need so see the different agar for example then I prefer face to face	4
2021/06/06 10:56:35 am GMT+3	Yes	Female	5	Year 4	60-90 minutes	Off Campus	N	N	N	N	N	N	N	N	N	N	N	N	COMBINATION of online and face to face sessions	Its better	3
2021/06/06 10:58:29 am GMT+3	Yes	Female	21	Year 4	60-90 minutes	Off Campus	N	A	D	A	A	D	A	D	SD	N	SA	N	COMBINATION of online and face to face sessions	Because of covid, itâ€™s better to not have all the sessions face to face, so I think a combination would be better, if the session needs seeing some specimens that are only available in the lab for example it can be face to face	4
2021/06/06 11:00:46 am GMT+3	Yes	Female	20	Year 3	60-90 minutes	Off Campus	D	A	A	A	D	D	D	D	N	N	N	N	FACE TO FCAE sessions only	Understanding much more	3
2021/06/06 11:01:22 am GMT+3	Yes	Female	21	Year 4	60-90 minutes	Off Campus	A	A	A	N	N	D	N	N	A	A	N	D	COMBINATION of online and face to face sessions	Material can be given online and then explained in face to face laby	4
2021/06/06 11:06:34 am GMT+3	Yes	Female	22	Year 3	60-90 minutes	Off Campus	A	SA	SA	SA	D	SD	SD	SD	A	SD	N	SD	FACE TO FCAE sessions only	more understanding and we can interact with each other	3
2021/06/06 11:08:13 am GMT+3	Yes	Female	21	Year 3	30-60 minutes	Both	SD	D	D	D	SD	D	D	D	SD	SD	SD	SD	FACE TO FCAE sessions only	-	4
2021/06/06 11:27:54 am GMT+3	Yes	Male	23	Year 4	less than 30 minutes	Off Campus	SA	SA	A	A	D	D	D	D	A	N	N	SD	COMBINATION of online and face to face sessions	The part of sessions in labs must be in f2f it enhance your focus on what you see and the theoretical part it can be both	2
2021/06/06 11:31:10 am GMT+3	Yes	Female	21	Year 3	30-60 minutes	Both	SD	D	D	D	SD	D	D	D	SD	SD	SD	SD	FACE TO FCAE sessions only	-	2
2021/06/06 11:31:30 am GMT+3	Yes	Female	21	Year 3	30-60 minutes	Both	SD	D	D	D	SD	D	D	D	SD	SD	SD	SD	FACE TO FCAE sessions only	-	2
2021/06/06 11:37:36 am GMT+3	Yes	Male	22	Year 4	60-90 minutes	Off Campus	A	SA	SA	D	A	A	A	A	A	SA	A	N	COMBINATION of online and face to face sessions	Dry labs should be online	2
2021/06/06 12:04:02 pm GMT+3	Yes	Female	22	Year 3	30-60 minutes	Off Campus	SA	SA	SA	SA	SD	SD	SD	SD	SA	SD	SD	SD	FACE TO FCAE sessions only	Online classes not that efficient to understand the subject	2
2021/06/06 12:22:58 pm GMT+3	Yes	Female	22	Year 3	60-90 minutes	Off Campus	N	N	N	N	SA	SA	SA	SA	SD	SA	SA	SA	ONLINE sessions only	--	3
2021/06/06 12:28:19 pm GMT+3	Yes	Female	22	Year 4	30-60 minutes	Off Campus	SA	N	N	D	SA	N	A	N	SD	SA	D	D	COMBINATION of online and face to face sessions	Online lecture first and then practical f2f to ensure understanding and be prepared to see lab content in real life with all information in head	3
2021/06/06 12:41:18 pm GMT+3	Yes	Male	24	Year 4	30-60 minutes	Both	SA	N	N	N	D	D	N	N	SA	SA	SA	SA	COMBINATION of online and face to face sessions	Honestly, Up to the number of the students because I believe most of the students are attending because they are mandatory, so better to be online with record which the student can go for it even more than once	5
2021/06/06 1:21:10 pm GMT+3	Yes	Female	22	Year 4	60-90 minutes	Off Campus	D	N	D	N	D	D	SD	N	N	D	D	D	COMBINATION of online and face to face sessions	To understand	5
2021/06/06 1:45:41 pm GMT+3	Yes	Female	21	Year 4	60-90 minutes	Off Campus	N	A	D	A	A	N	A	A	SD	N	SA	N	COMBINATION of online and face to face sessions	Because of covid, itâ€™s better to not have all the sessions face to face, so I think a combination would be better, if the session needs seeing some specimens that are only available in the lab for example it can be face to face	4
2021/06/06 1:52:45 pm GMT+3	Yes	Male	21	Year 3	60-90 minutes	Off Campus	SA	SA	SA	SA	SD	SD	SD	SD	SA	SD	D	SD	FACE TO FCAE sessions only	-	3
2021/06/06 2:00:20 pm GMT+3	Yes	Male	24	Year 4	30-60 minutes	Both	SA	N	N	N	N	N	N	N	SA	SA	SA	SA	COMBINATION of online and face to face sessions	Honestly, Up to the number of the students because I believe most of the students are attending because they are mandatory, so better to be online with record which the student can go for it even more than once	4
2021/06/06 2:03:45 pm GMT+3	Yes	Female	20	Year 3	60-90 minutes	Off Campus	A	D	D	D	SA	SA	SA	SA	SD	SA	SA	SA	ONLINE sessions only	the procedures done in the f2f labs are not always clear to all students since we are more than 200 students	3
2021/06/06 2:25:53 pm GMT+3	Yes	Female	21	Year 4	60-90 minutes	Off Campus	A	A	A	N	N	D	N	N	N	A	N	D	COMBINATION of online and face to face sessions	Material can be given online and then explained in face to face laby	4
2021/06/06 2:30:58 pm GMT+3	Yes	Female	21	Year 3	60-90 minutes	Off Campus	SD	N	SD	SD	SD	SD	SD	SD	SD	D	SD	D	COMBINATION of online and face to face sessions	combination between online and face to face sessions is better for understanding the concepts	5
2021/06/06 3:01:27 pm GMT+3	Yes	Female	22	Year 4	30-60 minutes	Off Campus	SA	N	N	D	SA	N	SA	N	SD	SA	D	D	COMBINATION of online and face to face sessions	Online lecture first and then practical f2f to ensure understanding and be prepared to see lab content in real life with all information in head	5
2021/06/06 3:12:29 pm GMT+3	Yes	Female	23	Year 4	30-60 minutes	Off Campus	A	A	SA	A	D	SD	SD	D	D	SD	SD	SD	FACE TO FCAE sessions only	Better in interaction between doctors and students	4
2021/06/06 3:25:52 pm GMT+3	Yes	Female	22	Year 4	60-90 minutes	Off Campus	A	A	A	N	N	D	N	N	N	SA	N	SD	COMBINATION of online and face to face sessions	Sometimes we need to see the slides under the microscope, and other things we need to see it by ourself, thatâ€™s why the online classes are important, but I believe that the online sessions can be beneficial for the solid material, for example we can take the material by an online session and the next day we attend the l[illegible]	3
2021/06/06 3:33:05 pm GMT+3	No																				
2021/06/06 6:48:52 pm GMT+3	Yes	Male	22	Year 3	30-60 minutes	Both	D	D	D	D	D	D	D	D	D	D	D	D	COMBINATION of online and face to face sessions	-	3
2021/06/06 8:07:04 pm GMT+3	No																				
2021/06/07 2:24:37 pm GMT+3	Yes	Female	20	Year 3	60-90 minutes	Off Campus	A	N	A	D	N	A	N	A	D	A	A	N	COMBINATION of online and face to face sessions	Saves time	4
2021/06/07 5:38:29 pm GMT+3	Yes	Female	21	Year 3	30-60 minutes	Off Campus	A	A	A	D	A	N	A	A	D	SA	A	A	COMBINATION of online and face to face sessions	To provide more spectrum to help with understanding the lesson	4
2021/06/08 12:40:46 am GMT+3	Yes	Male	21	Year 4	60-90 minutes	Off Campus	A	N	A	D	SA	N	A	SA	D	A	A	D	COMBINATION of online and face to face sessions	Some of the lab sessions must be in the lab but not all of them, so a combination would be the best option in my opinion.	5
2021/06/08 8:16:33 am GMT+3	Yes	Female	23	Year 4	30-60 minutes	Off Campus	A	A	SA	A	D	SD	SD	D	D	SD	SD	SD	FACE TO FCAE sessions only	Better in interaction between doctors and students	4
2021/06/08 11:03:12 am GMT+3	Yes	Female	24	Year 4	60-90 minutes	Off Campus	D	D	D	D	SA	SA	SA	SA	SA	SA	SA	SA	ONLINE sessions only	Easy to understand and repeat it when i want	3
2021/06/08 11:53:58 am GMT+3	Yes	Female	21	Year 4	60-90 minutes	Off Campus	N	N	A	N	N	N	D	D	A	A	A	N	COMBINATION of online and face to face sessions	Itâ€™s important to observe with your eye rather than only memorizing pictures	4
2021/06/08 6:09:22 pm GMT+3	Yes	Female	21	Year 3	60-90 minutes	Off Campus	D	D	D	D	A	A	A	A	D	SA	N	SA	ONLINE sessions only	We able to repeat the class as many times as we want	4
2021/06/08 11:32:12 pm GMT+3	Yes	Male	21	Year 4	less than 30 minutes	Off Campus	SA	SA	SA	SA	D	D	N	A	N	N	N	SD	COMBINATION of online and face to face sessions	Face to face will be enhanced by online additional sessions	4
2021/06/08 11:33:55 pm GMT+3	Yes	Female	20	Year 3	30-60 minutes	Off Campus	SA	SA	SA	A	N	A	N	SA	SA	A	A	D	COMBINATION of online and face to face sessions	because some lab topics have some much background that it is easier to have the lecture at home but the practical f2f	4
2021/06/09 11:00:32 am GMT+3	Yes	Male	21	Year 3	30-60 minutes	Off Campus	SA	SA	SA	SA	D	SD	N	N	SA	SD	D	SD	FACE TO FCAE sessions only	For better understanding	5
2021/06/09 7:36:36 pm GMT+3	Yes	Female	24	Year 4	60-90 minutes	Off Campus	SA	SA	SA	SA	D	D	D	SD	A	A	N	SD	FACE TO FCAE sessions only	More interaction and understanding	4
2021/06/10 12:00:28 am GMT+3	Yes	Female	22	Year 4	30-60 minutes	Off Campus	SA	A	A	D	A	N	A	A	SA	SA	A	N	COMBINATION of online and face to face sessions	some subjects need the face to face component while others just need the explaining through online resources	4
2021/06/10 2:33:04 am GMT+3	Yes	Female	21	Year 4	60-90 minutes	Off Campus	SA	SA	SA	A	SD	A	A	A	N	SA	SD	SA	COMBINATION of online and face to face sessions	-	3
2021/06/10 4:23:13 am GMT+3	Yes	Female	21	Year 3	60-90 minutes	Off Campus	A	A	A	A	N	N	N	N	N	N	N	N	ONLINE sessions only	It takes less time	2
2021/06/10 12:39:38 pm GMT+3	Yes	Male	21	Year 3	30-60 minutes	On Campus	A	N	A	N	N	N	N	D	N	A	A	D	COMBINATION of online and face to face sessions	To improve microbiology lab	3
2021/06/10 1:11:40 pm GMT+3	Yes	Female	20	Year 3	30-60 minutes	Off Campus	SA	SA	SA	SA	SD	SD	SD	SD	SA	SD	D	SD	FACE TO FCAE sessions only	It is more beneficial for us as medical students to attend classes face to face	3
2021/06/13 10:09:13 am GMT+3	Yes	Male	22	Year 4	30-60 minutes	Off Campus	A	SA	A	A	SD	SD	D	D	D	D	D	D	FACE TO FCAE sessions only	Micro needs to be done handly not only watching	4
2021/06/13 12:25:39 pm GMT+3	Yes	Female	23	Year 4	30-60 minutes	Off Campus	A	A	A	D	A	N	N	N	D	N	A	D	COMBINATION of online and face to face sessions	-	4
2021/06/13 8:21:15 pm GMT+3	Yes	Male	21	Year 4	30-60 minutes	Off Campus	A	SA	SA	A	N	SD	SD	N	A	N	A	SD	FACE TO FCAE sessions only	Helps students to focus more on the subject and gives them the chance to interact with the tutor and the equipment around them	4
2021/06/15 2:34:06 pm GMT+3	Yes	Female	20	Year 3	60-90 minutes	Off Campus	A	A	SA	SA	SD	SD	SD	A	SA	D	A	SD	COMBINATION of online and face to face sessions	Itâ€™s good to go and see for yourself f2f however it is also nice to have something online to go back to for revision	5
2021/06/15 3:52:18 pm GMT+3	Yes	Female	22	Year 4	30-60 minutes	Off Campus	D	D	D	D	SD	D	SD	N	N	N	N	N	COMBINATION of online and face to face sessions	Better interaction in face to face and time saving in online	3
2021/06/15 3:57:27 pm GMT+3	Yes	Male	23	Year 4	60-90 minutes	Both	D	N	A	N	N	N	N	N	N	N	N	N	COMBINATION of online and face to face sessions	Sometimes we have a small lab like 30 min or less the online will be good or sometimes	3
2021/06/15 7:25:21 pm GMT+3	Yes	Female	22	Year 3	30-60 minutes	Both	SD	SD	A	D	D	A	A	A	A	A	A	A	COMBINATION of online and face to face sessions	-	5
2021/06/15 7:34:27 pm GMT+3	Yes	Female	20	Year 3	60-90 minutes	Off Campus	A	A	SA	SA	SD	SD	SD	A	SA	D	A	SD	COMBINATION of online and face to face sessions	Itâ€™s good to go and see for yourself f2f however it is also nice to have something online to go back to for revision	5
2021/06/16 2:48:55 am GMT+3	Yes	Female	22	Year 4	30-60 minutes	Off Campus	D	D	D	D	SD	D	SD	N	N	N	N	N	COMBINATION of online and face to face sessions	Better interaction in face to face and time saving in online	3
2021/06/16 6:52:35 am GMT+3	Yes	Female	23	Year 4	30-60 minutes	Both	N	N	N	N	N	N	N	N	N	N	N	N	COMBINATION of online and face to face sessions	to be more clear	3
2021/06/16 8:53:37 am GMT+3	Yes	Female	23	Year 4	30-60 minutes	Both	N	N	N	N	N	N	N	N	N	N	N	N	COMBINATION of online and face to face sessions	to be more clear	4
2021/06/18 12:44:35 pm GMT+3	Yes	Male	21	Year 3	60-90 minutes	Both	SA	SA	SA	SA	SD	SD	SD	SD	SA	SD	N	SD	COMBINATION of online and face to face sessions	Because we are medical students!!!!!	4
2021/06/21 8:56:09 am GMT+3	Yes	Male	21	Year 3	60-90 minutes	Off Campus	SA	A	SA	N	A	A	A	A	N	SA	SA	N	COMBINATION of online and face to face sessions	Skills achieved by face to face labs	4
2021/06/21 9:02:53 am GMT+3	Yes	Male	20	Year 3	30-60 minutes	Off Campus	SA	N	A	N	N	N	A	A	A	SA	SA	N	COMBINATION of online and face to face sessions	Practical sessions are needed for medical students	5
2021/06/21 9:11:57 am GMT+3	Yes	Male	21	Year 3	60-90 minutes	Off Campus	A	N	A	N	N	N	A	A	N	SA	SA	N	COMBINATION of online and face to face sessions	medical students needs practical labs not online labs	4
2021/06/21 6:08:37 pm GMT+3	Yes	Female	22	Year 3	30-60 minutes	Both	SD	SD	A	D	D	A	A	A	A	A	A	A	COMBINATION of online and face to face sessions	-	4
2021/06/23 12:11:48 pm GMT+3	Yes	Female	21	Year 3	60-90 minutes	Off Campus	N	A	A	N	SA	N	N	N	D	D	N	D	COMBINATION of online and face to face sessions	The practical session should be face 2face and the theory should be online	4
2021/06/24 11:44:46 am GMT+3	Yes	Male	21	Year 3	30-60 minutes	Off Campus	A	N	SA	N	A	N	N	N	N	SA	SA	N	COMBINATION of online and face to face sessions	face to face is better	5
2021/06/27 1:22:12 am GMT+3	Yes	Female	21	Year 3	60-90 minutes	Off Campus	N	A	A	A	SA	N	A	N	D	D	N	D	COMBINATION of online and face to face sessions	The practical session should be face 2face and the theory should be online	4
2021/06/27 12:46:48 pm GMT+3	Yes	Female	20	Year 3	60-90 minutes	Off Campus	A	A	A	N	SA	SA	SA	SA	D	SA	N	N	COMBINATION of online and face to face sessions	Bec the material which are doctors only presents on the presentations are more understandable online than face to face and the videos or in general the material which need some work it is more understandable face to face	5
2021/06/28 9:34:04 am GMT+3	Yes	Male	22	Year 3	less than 30 minutes	Off Campus	D	D	D	D	SA	SA	SA	SA	SA	SA	SA	SA	ONLINE sessions only	Online session can cover all the materials needed in a much sufficient way in comparison with f2f sessions.	4
2021/06/28 10:38:42 am GMT+3	Yes	Male	21	Year 3	30-60 minutes	Off Campus	SA	SA	SA	N	A	N	A	N	A	SA	SA	N	COMBINATION of online and face to face sessions	medical students should know through hands on labs which is better to understand.	5
2021/06/29 10:17:35 am GMT+3	Yes	Male	21	Year 3	60-90 minutes	Off Campus	SA	SA	SA	N	A	N	A	N	N	SA	SA	SD	COMBINATION of online and face to face sessions	WE LEARN SKILLS BY DOING THE TESTS AND NOT BY WATCHING VIDEO	4
2021/06/29 10:20:20 am GMT+3	Yes	Male	21	Year 3	60-90 minutes	Off Campus	A	N	A	N	A	N	A	A	A	SA	SA	N	COMBINATION of online and face to face sessions	all the practical sessions should be face to face for better understanding and theoritical or case discussion can be online.	5
2021/06/29 10:00:16 pm GMT+3	Yes	Female	21	Year 3	60-90 minutes	Off Campus	N	A	A	N	SA	N	A	N	D	D	N	D	COMBINATION of online and face to face sessions	The practical session should be face 2face and the theory should be online	4
2021/07/06 12:37:29 am GMT+3	Yes	Female	21	Year 3	60-90 minutes	Off Campus	D	D	D	D	A	A	A	A	D	SA	N	SA	ONLINE sessions only	We able to repeat the class as many times as we want	5
2021/07/30 1:26:26 pm GMT+3	Yes	Female	23	Year 4	60-90 minutes	Off Campus	N	N	N	N	N	N	N	N	N	N	N	N	COMBINATION of online and face to face sessions	-	[illegible]

Footnote: SD - Strongly Disagree, D - Disagree, N - Neutral, A - Agree, SA - Strongly Agree
