# Supplementary material for: Perception of online and face to face microbiology laboratory sessions among medical students and faculty at Arabian Gulf University: a mixed method study
Source: BMC Med Educ. 2022 May 30;22:411. doi: 10.1186/s12909-022-03346-2 (PMC9149330; doi:10.1186/s12909-022-03346-2)
Supplement: Supplementary file 5 — Additional file 5. [file 12909_2022_3346_MOESM5_ESM.pdf]

STUDENT QUESTIONNAIRE

This survey is to discern the perception of Online and Face to Face(f2f) microbiology laboratory sessions among medical students at Arabian Gulf University.

Please indicate your level of agreement or disagreement with the following statements by ticking one of the answers for each statement.

Possible risks and benefits to the participants: there are no anticipated risks related to participating in this study. You may need to spend 10 to 15 minutes for answering this survey. The possible long term benefit might be the revision of microbiology lab curriculum implementation which could possibly improve student and faculty satisfaction.

Please note that your participation in this study is entirely voluntary and you always have the right to withdraw from the study at any point of time.

This is an anonymous survey. Any information that is obtained during this study will be kept confidential and will be destroyed after the completion of the study.

Ethical considerations:
This study is approved by Research and Ethics committee of Arabian Gulf University.

---

*Required

1. I give my consent to participate in this survey *

   Mark only one oval.

   - ⬭ Yes
   - ⬭ No

Part 1

PERSONAL PROFILE

2. 1. Gender *

   Mark only one oval.

   - ⬭ Male
   - ⬭ Female

3. 2. Age in years *

____

4. 3. Year of study *

Mark only one oval.

- Year 3
- Year 4

5. 4. What amount of time did you spend on each online practical? *

Mark only one oval.

- less than 30 minutes
- 30-60 minutes
- 60-90 minutes

6. 5. Where did you work on the online practicals? *

Mark only one oval.

- On Campus
- Off Campus
- Both

Part 2 Your perceptions about the face to face (f2f) microbiology lab sessions.

Please select the appropriate option for each item based on your opinion, SD - Strongly Disagree, D - Disagree, N - Neutral, A - Agree, SA - Strongly Agree *

7. SD - Strongly Disagree, D - Disagree, N - Neutral, A - Agree, SA - Strongly Agree * *

Mark only one oval per row.

	SD	D	N	A	SA
The f2f labs were enjoyable					
I preferred the f2f labs					
f2f labs enhanced my understanding of the course					
All labs should be f2f					

Part 3 Your perceptions about the online microbiology lab sessions.

Please select the appropriate option for each item based on your opinion, SD - Strongly Disagree, D - Disagree, N - Neutral, A - Agree, SA - Strongly Agree *

8. SD - Strongly Disagree, D - Disagree, N - Neutral, A - Agree, SA - Strongly Agree * *

Mark only one oval per row.

	SD	D	N	A	SA
The online labs were enjoyable	⬭	⬭	⬭	⬭	⬭
I preferred the online labs	⬭	⬭	⬭	⬭	⬭
Online labs enhanced my understanding of the course	⬭	⬭	⬭	⬭	⬭
The different types of interaction (animations etc.) in the online labs improved my learning.	⬭	⬭	⬭	⬭	⬭
I found it difficult to follow the flow and meaning of the subject material in the online labs	⬭	⬭	⬭	⬭	⬭
I consider the online labs to be an essential addition to traditional f2flabs	⬭	⬭	⬭	⬭	⬭
Technical enablement (internet/software/hardware) of online sessions was satisfactory	⬭	⬭	⬭	⬭	⬭
All labs should be online	⬭	⬭	⬭	⬭	⬭

Part 4 Opinion on preferred method

9. 1. The best option to deliver microbiology lab sessions would be through one of the following *

Mark only one oval.

- ⬭ ONLINE sessions only
- ⬭ FACE TO FCAE sessions only
- ⬭ COMBINATION of online and face to face sessions

10. 2. Give reasons for your response to the above question *

11. 3. Compared to other AGU medical school learning experiences, to what extent do you feel that the microbiology lab sessions added value to the MD curriculum? Select on a scale of 1 to 5, where 1 - no value, 5 - very high value *

Mark only one oval.

	1	2	3	4	5	
No value	⬭	⬭	⬭	⬭	⬭	Very high value
