# Supplementary material for: Perception of online and face to face microbiology laboratory sessions among medical students and faculty at Arabian Gulf University: a mixed method study
Source: BMC Med Educ. 2022 May 30;22:411. doi: 10.1186/s12909-022-03346-2 (PMC9149330; doi:10.1186/s12909-022-03346-2)
Supplement: Supplementary file 6 — Additional file 6. [file 12909_2022_3346_MOESM6_ESM.pdf]

Dr. Archana:

All of you should press continue if you see this in your screen. So, should we call the other student, Ronny, who is not there? Or shall we start?

Dr. Ronny:

So, we will start and maybe she can join in.

Dr. Archana:

Okay no problem, you start first.

Dr. Ronny:

A very good morning to all of you and we welcome you today for our focus group discussion session. Myself, Dr. Ronny I am one of the investigators of this project, and I have my colleague with me, that's Dr. Archana from the Department of Medical Education who is also a co-investigator in this project. So, this focus group discussion is conducted as part of the study titled, "The Perception of Online and Face-to-Face Microbiology Laboratory Sessions Among Medical Students and Faculty at AGU." So, thank you all once again for giving your informed consent for taking part in this discussion and please feel free to share your views about your experiences with online as well as face-to-face microbiology laboratory sessions at AGU.

So, this study is approved by the Research and Ethics Committee, AGU numbered E049PI4/21 and this discussion will be video recorded to ensure that we do not miss any of your valuable comments. So, it would last for around 20 to 30 minutes and all the contents of the discussion will be maintained confidential and your identity will not be revealed in any manner and the transcribed files will be kept in a secure location and will be destroyed after completion of this study. So, please feel free to express your thoughts and we will try our level best to take your valuable feedback into account for our future microbiology lab sessions. Thank you again. So, let's begin, doctor?

Dr. Archana:

Thank you, Ronny. As Ronny made it very clear, the main objective of this project is to reflect upon our experience and see what best we can do in future. So, nothing is perfect, you know, we have to reflect and learn from our mistakes, and we have to improve our

teaching-learning methods especially in the field of medicine. It is very important that we make changes, we adjust, and important stakeholders in the process is students. Ultimately, it is you who have to tell us whether it was working or not working, and it is you the young minds that should give us ideas and different suggestions to make it better. So, with this I will start today's session. Please feel free to express yourself, let it be an informal discussion. Just don't worry about your identity as Dr. Ronny said, nothing will be revealed at the end of the session.

Okay the first question is, can you please describe your experience with online microbiology lab session, with focus on online microbiology lab session? Can you describe your experience please?

Adhup:
Hi doctor, do you want us to do in order alphabetically, or do you want us to just speak whoever wants to speak?

Dr. Archana:
Whoever wants to speak can start, if anyone else has to interrupt just raise your hand we will give you chance. You know how to raise your hand? In reactions if you click there is an option called raise your hand? And we will allow you to talk.

Adhup:
Alright.

Dr. Archana:
You can go ahead Adhup, yes.

Adhup:
Okay doctor. Personally, for me I found that when the lab sessions were online because usually the microbiology they last about one hour, was very difficult to keep focus for the whole duration. Meanwhile when it was in person it was a lot easier for us to keep intact and pay attention to what the doctor is saying. And this is why I don't know even with the interactions like the doctors try to make us interact, but we wouldn't interact as much as we would when we were in person, because of the lack of focus. It was a lot easier to get distracted. That was my personal experience with (0:05:00) the microbiology. Like I would

focus at the beginning start and then by the end I would have zoned off several times to the point where I wouldn't know what the doctor is talking about anymore.

Dr. Archana:

Anyone else who would like to add to what Adhup said?

Razan:

Yes, I want to, I agree with Adhup. And also, I know you doctors did your best, but still sometimes too much because the new information in person like we go to two sessions because we often are two groups – so we switch labs. So, maybe easier in face-to-face than online.

Dr. Archana:

Thank you Razan. Noor, yes?

Noor:

In online it is bit disengaging like in real lab it used to be much easier to ask questions like, I build memories with the knowledge I take and in online it is just staring at the screen and the slides are all similar, so it is hard to stay focused.

Dr. Archana:

Thank you, Noor. Anyone else? Amina, you want to talk, yes please go ahead.

Amina:

Yes doctor, I totally agree with them, and I want to add something that maybe when we come to the labs and face-to-face classes, sometimes we get to sit at the back of the lab so we don't hear the doctor properly or few students at the back are talking and making distractions so, online maybe for discussion only or as a theory slides it is better online.

Dr. Archana:

Thank you, Amina. Okay Badar also agrees with same thing. Okay, Jumana your mic is on you want to talk something?

Jumana:

Yes doctor, I agree with Amina and personally I think that I could learn this subject in online lab session. And I prefer to attend on time rather than studying from record, but the record is helping us because we can make sure that we get all the information, and we can learn as much as possible.

Dr. Archana:
Thank you, Jumana. Now we move on to our next question. In your opinion what are the advantages of this online microbiology session? Do you think were there any advantages if yes, what were they? Can you please tell us?

Adhup:
Can I speak doctor?

Dr. Archana:
Yes, yes, Adhup please.

Adhup:
Just like Amina mentioned with the online for theory things we manage to catch all of the information even if we don't listen in the session, we can listen later on. This is probably the most advantage of online, that regardless sometimes people that sit on the back can't listen or we have to come early in order to get seat in the front, in order to listen properly.

Dr. Archana:
Thank you, Adhup. Anyone else would like to add any advantages of online?

Badar:
Hello can I speak?

Dr. Archana:
Yes, Badar please.

Badar:
Because I do not see the raise hand emoji but okay.

Dr. Archana:

That’s okay.

Badar:

I see that online experience is much time saving. It saves for us a lot of time. But as my colleague said it is hard to stay focused for a prolonged period of time, but when we were at the university we used to move between sessions, so there was dynamic, so you can refresh yourself and so on. But for the record even if we don’t catch everything during the online session, we can catch it later on. So, there is no problem with gaining information, but the problem is for staying focused I think, during the session. But the advantage is one of the really most important one is time saving for me.

Dr. Archana:

Thank you. Yes?

Adhup:

Just add to what Badar said about the time saving (0:10:00) and the time. Noor mentioned something previously, Noor said about the fact that she builds memories from what she hears or what she sees in person, and I think a huge distinguishing factor between labs and actual lectures is the fact that labs are practical, that’s something you see in front of you, and it is more of an experience rather than just obtaining lab knowledge. And by having it online I personally feel like that experience aspect is completely demolished. And because of that we probably need to work, double the amount of work in order to keep this information in our heads. And this is a point that not a lot of people take into consideration with the online schooling. So, yes, I just wanted to add to what he said about that although it has its advantages but in the end result this is a lab.

Dr. Archana:

Thank you Adhup. Jumana your hand is raised is it from previous question or you are raising it again? Jumana?

Jumana:

No, I raised again. Also, one of the advantages is the atmosphere is more quieter, learning it up, and the voice is more clear for the students than the face-to-face lab session.

Dr. Archana:

Thank you Jumana. Now we go to the next question which is opposite of what I asked before. What are the disadvantages you faced in online microbiology session? What are the things you didn't like about online microbiology lab session? Was there anything you didn't like about online lab, or was there any difficulty?

Badar:

Doctor, for me it was sometimes technical issues to be honest, sometimes, not all the time.

Dr. Archana:

Okay, thank you Badar. Anyone else did you feel any difficulty with online lab session?

Adhup:

Doctor just like we mentioned previously, when we were in person it was very easy for us to stop and ask the doctor whether it was after the class or before the class. Just having that advantage, it actually makes the student feel a lot more confident about the knowledge they are obtaining from the session. And I know on online we can do that however some people are shy and because they are very shy, they don't want to ask the doctor in front of other people and that creates a lot of learning barriers for those students especially. Some people aren't but most of the students don't want to stop the doctor in the middle of the lecture and ask them well what was that. But just like we mentioned previously with the switching, for example, from one lab to another, the students get the opportunity even if it is for few seconds just to clarify a point or two, or after the sessions they can immediately just go to the doctor get the information cleared out. That's a lot easier for them.

Another disadvantage was seeing a slide, or seeing a smear, or seeing the process being done in front of you is a lot different from us seeing it via a video. We already do that, whether it was before when we were in person or when we were online, we can YouTube whatever you taught us if it wasn't clear and look up the video. Hence it just completely removes experience of trying to keep the information remaining in our heads and I think that's probably the biggest disadvantage. Yes, we have the advantage of saving time, yes, we have the advantage of being able to pick up every single word, but at the end of the day, we are here to become the best doctors we are, not only to save the time we need to save.

Dr. Archana:

Thank you Adhup. Jumana, you want to say something? Now we shift our focus from online to face-to-face sessions. Can you describe your experience with face-to-face lab sessions in microbiology? All of you would have attended some sessions, right? How was your experience in face-to-face, how would you like to describe it? (0:15:00)

Badar:
It was great for me, because I had to interact more with my colleagues and with the doctors, it would make the information more clear. But as we said before, one of my colleagues said sometime the seat they got full, so I sit in back there is a noise. So, online is better in that aspect of that quietness and clear screen but yes, that was my point about the seat sometimes it gets full very early.

Dr. Archana:
Thank you Badar. Zainab your hand is raised.

Zainab:
Thanks doctor. I agree with them about what they said. The labs were very much enjoyable and they were interactional and as you've been seeing a lot of the information is best received afterwards. But maybe the main issue that's repeated a couple of times was the amount of slides. Sometimes like some sessions take more time than others and sometimes some slides are left that the doctors are not able to finish thoroughly. And so, a couple of times we had maybe 10 slides gone over in 2 minutes. However, in the online session almost always the doctors have more time and if they don't, they can also give us extra sessions or explain their points to the point.

Dr. Archana:
Thank you, Zainab. Noor you can speak now.

Noor:
I completely agree. Sometimes it's felt that the doctor was rushing to be on time, because they will have to switch sessions, and that usually cause like when he presents, we also feel the stress or the worry the energy that the doctor is giving, so, it used to be stressful. I remember our last microbiology lab in the morning hour we had 100 slides and I think we only finished in the lab 30 so it wasn't like we did not cover all of the objectives; it was rushed, and the timing was so tight, and we had resources after it directly. So, I remember

that it was stressful. Online it is much more relaxing, you are at home, you are relaxed, that was one of the things that is a disadvantage with face-to-face labs.

Dr. Archana:
Thank you, Noor. Just to summarize, can you tell me few important advantages of face-to-face in a lecture, very important advantage of face-to-face sessions? Amina, yes?

Amina:
Advantages of being at the lab is working with the materials, like we can do the slides or view it in the microscope, which makes it more fun, and information stays in your mind. That's it.

Dr. Archana:
Thank you, Amina. Hessa?

Hessa:
I agree with Amina because with this I can work with my hand, so I can learn easier and also, I can ask my questions directly to the doctor. So, that aspect was very important to me. While in the online some of the questions I couldn't really explain my perspectives to the doctor by email only. So, that was a little hard for me.

Dr. Archana:
Okay, thank you. Yes?

Adhup:
Doctor can I just...

Dr. Archana:
Yes, yes, Adhup.

Adhup:
I think the quality of knowledge on face-to-face is a lot higher than online. In online it is easier to obtain, and it is more efficient however, with the face-to-face the quality is a lot higher and if there is anyone who disagrees with that you can mention that, like to my colleagues I am speaking.

Dr. Archana:

Okay. Thank you, Adhup. Yes, Razan?

Razan:

Yes, I just wanted to say like I agree with Adhup. For example, in real life when you are studying like the type of culture okay after that we will see it, not just a picture. So, it is much better face-to-face.

Dr. Archana:

Thank you.

Badar:

I would like to add something little.

Dr. Archana:

Yes, Badar. (0:20:00)

Badar:

To take into consideration, we are now focusing on the learning process but what about the conditions. We are not having all the same conditions, during this period, this pandemic. So, even though the face-to-face has higher quality in terms of learning and interaction but what is the more suitable method during this period, is what matters. Some people want to go to AGU but maybe their families are refusing. Just as a thought, whereas other families are okay with that. So, maybe it is an important thing to consider.

Dr. Archana:

Yes, thank you Badar for adding your point. Anyone else for advantages of face-to-face? Now can you quickly tell the major disadvantages of face-to-face lab sessions? The major ones which you want to share.

Adhup:

Honestly doctor I think we already mentioned the numbers and the seats. And if it was in person and we were to come back I think the numbers would be already reduced. So I think

that might already eliminate our problem which is that we can’t find seats or the noise. But I think what everyone discussed was the seating arrangement.

Dr. Archana:
Okay, the seating arrangement, the noise. Yes, Zainab you want to add something?

Zainab:
Yes doctor. Another thing is that I know that we are supposed to self-learn and get our information by ourselves but sometimes that we like to go over the slides and often they are all over the place. And there are also a lot in number, and they are all over the place in which the doctor goes back and forth, two slides forward, two slides backwards, where we can't just study, we can’t do that. And sometimes a picture of one organism is in between another organism, so we have to rearrange everything again. So, maybe if someone could go over the slides and just have a look at them that would be very helpful for us.

Dr. Archana:
This is in face-to-face or? Ronny did you understand was it the context for face-to-face?

Zainab:
Yes.

Dr. Ronny:
I think this is – Zainab is talking about face-to-face, right?

Zainab:
Yes.

Dr. Ronny:
The slides what they put on the drive.

Zainab:
Yes, the one that you put on the big screen.

Dr. Ronny:
Thank you.

Dr. Archana:

Thank you. Now we have heard discussion of both advantage and disadvantage of both online and face-to-face. Now we know both the sides of both the approaches, my next question would be, do you think blended learning, you know what I mean by blended learning? The combination of online session with the face-to-face session blended, something from here and something from there. Do you think this blended learning approach will suit microbiology lab session? Yes Zainab?

Zainab:

Yes doctor. I believe it is suitable because sometimes some sessions are not practical, they are just slides. So, we have for example, first session two of them are practical and then two the doctor is explaining off the slides, so, I think that these could be still online. Another thing is that's when we have short sessions, for example, our labs sometimes are not very long, 15 minutes or half an hour, and that would also be practical to be online so that we don't have to come all the way just for 15 minutes.

Dr. Archana:

Thank you, Zainab. Razan?

Razan:

I agree with Zainab. But also, like let's be real. Nothing will return 100% after COVID. So, I think combination between them will be good, because maybe like we are a year and a half now on the online sessions, so it will be like a little difficult to switch to face-to-face. That's it.

Dr. Archana:

Okay, thank you. Noor? Noor your hand is raised.

Noor:

I believe since we tried both face-to-face and online, we should take the advantages from both and try to make the best of it. So, as we all mentioned that the resource session (0:25:00) it is much better to have them online but like the practical as Zainab said we would rather have them in the university where we could have the ability to ask the doctor.

We don’t want like microbiology labs that are two hours long, if it is only 30 minutes where we could see the practical things and engage with our doctor that would be like ideal.

Dr. Archana:

Thank you, Noor. Adhup, yes?

Adhup:

Hi, doctor. I agree with Noor with her statement, especially the lab sessions should be in person, because I think it is a lot more effective. But in terms of the resource sessions, they can be online. We don’t mind the resource sessions being online. However, for lab it is just to consolidate all information I think it is lot better to have them in person.

Dr. Archana:

Thank you. I have just one more question for all of you. In microbiology there are few topics, I think Ronny will know better. There are few topics like sample handling, processing, recording, and interpreting the results. So, these sessions, you know, handling of the samples, how to process the sample, how to interpret the results, these kinds of sessions what do you think should be the methodology? Do you think online is better or face-to-face? How should we schedule these sessions? Ronny, can you add more about these topics, are they purely theory or do they have practical?

Dr. Ronny:

Practical yes, especially when it comes to sample handling, I think we just discussed with them how do we handle the samples and all. But processing is very important wherein we show how its culture is being done or biochemical test or serological test how it is being conducted, and interpretation of the results, so that is also important wherein you get the report and how do you interpret it. So, based on this, what do you feel a blended session or face-to-face or online, which is better?

Dr. Archana:

For these three topics sample handling, processing, and interpretation of the discovery. Yes, Adhup.

Adhup:

Personally speaking doctor for me in online with the slides this part specifically was the most difficult to study for. And that was because even though you try your best to show us the videos sometimes they are not clear. Or like Badar mentioned there is technical difficulties with the sound or the video sometimes it is because of the internet, they don't show fully. Personally, I have the most difficult time to study these topics and I think that's why I probably have to go on Google or watch a video on YouTube in addition to that which wastes even more of my time. And these videos usually don't show us what the university wants specifically from us. I feel like having these specific sessions in person makes it a lot easier especially with the slides, because most of the slides are very long and they have a lot of details, like numbering and things like that. I understand that this is just to make sure that we have the full picture, however sometimes these details are not as important as other details in the slides. So, yes that's all for me.

Dr. Archana:
Thank you Adhup. Anyone else for these sessions, what are your opinions? Sample handling...

Badar:
Yes, I would like to add something.

Dr. Archana:
Yes Badar.

Badar:
Yes, for the blended learning, I think what you mean by blended, the main is, that the resource sessions, the slides, the theory part, it is okay to be online. But about the practical part, which is the sampling, handling, etc., I think it would be better to be practical at university in combination with the review for microbiology. So, we can take the chance to view the process face-to-face and at the same time if we have questions, we can ask them directly whatever we want in that session. I think that's my opinion about blended learning.

Dr. Archana:
Thank you Badar. Anyone else to wrap up any other thing which we have not asked you want to convey? These are the questions from our side, as students you want to say

something which we have not covered, or you want to share (0:30:00) something or you want to give some suggestion?

Zainab:
Doctor, we just want to thank you all for your efforts during this time. Because we know it was difficult for all of us so we really appreciate the fact that you want to know more about how we feel and hopefully these things can be implemented.

Dr. Archana:
Thank you, Zainab, thank you so much. Anyone else who want to say something which we have not covered in these questions, you think that could be addressed? Anything? Yes, Adhup, yes?

Jumana:
I think I have heard with online and face-to-face points we wanted to like say about.

Dr. Archana:
Okay. Adhup yes?

Adhup:
Yes doctor, I just think the last thing I just wanted to mention was the point about the slides. I found that even the slides during online I think for one of the especially in the GI system, some of the slides we had were not very clear and they had a lot of things that were like from external resources that weren't very specific about what we needed to know. And I think that's had us a lot of surprise in the exam even with the questions. They were relatively little bit more difficult than other sessions. The slides weren't very clear as well. So, I think the slide problem I personally faced with both face-to-face as well as online. Face-to-face it was the going back and forth just like Zainab mentioned. However, with online it was just the quality of the slides weren't efficient enough for us to know exactly what you want from us, for this specific. Because I know you are aware doctor that sometimes we do the test with an organism for example in year 2 and then we come back to it again in year 3. So, sometimes the extent of the information we need to know about this organism for this specific exam isn't very clear. That's pretty much it. And thank you doctors for having us and listening to our opinions, we really do appreciate it.

Dr. Archana:

Thank you. We would like to thank each and every one of you for taking your time and believe us we will try to make our best. We will take all your opinions, we will listen to this interview several times, we have to take notes, we will consider, and we will put forth the recommendations to the university. And whatever is within our limits we will try to implement it as early as possible. And whatever we need permission from the Deans and other administrators we will also try to do it as early as possible. Thank you very much, thanks a lot. Ronny?

Dr. Ronny:

Yes, actually we really thank all of you for attending this session. Thank you all for your valuable inputs in this session.

Dr. Archana:

Thank you, have a great day. Wish you all the best.

Zainab:

Have a good day.

Noor:

Thank you, bye.

Adhup:

Thank you doctors, have a nice day.

Dr. Archana:

Yes you too. Thank you.

Jumana:

Thank you.
