# Supplementary material for: Perception of online and face to face microbiology laboratory sessions among medical students and faculty at Arabian Gulf University: a mixed method study
Source: BMC Med Educ. 2022 May 30;22:411. doi: 10.1186/s12909-022-03346-2 (PMC9149330; doi:10.1186/s12909-022-03346-2)
Supplement: Supplementary file 7 — Additional file 7. [file 12909_2022_3346_MOESM7_ESM.pdf]

Dr. Ronnie:

Good morning, Maria.

Maria:

Good morning, doctor.

Dr. Ronnie:

Hello Madhavi, good morning, dear.

Madhavi:

Hi doctor, good morning.

Dr. Ronnie:

How are you all doing?

Madhavi:

We're all good, thank you.

Dr. Ronnie:

Hello, Noah, how are you?

Female Speaker:

Hi, doctor, fine.

Dr. Ronnie:

Hello. Okay, okay, no, sure, sure. Hi, Sara and Zahra.

Female Speaker:

Hi, doctor.

Female Speaker:

Hi, doctor. Good morning.

Dr. Ronnie:

Good morning. Good morning. So six students have joined us?

Female Speaker:

Yeah. So we're expecting one more.

Dr. Ronnie:

Okay, good. Hi guys. Good morning.

Female Speaker:

Hello doctor.

Female Speaker:

Hi, doctor.

Female Speaker:

Good morning, doctor. (0:05:00)

Dr. Ronnie:

Okay, good morning Amina. Ronnie. Are we waiting for any more? Okay, Prof. Shahid was - one second. Okay, so we'll start, doctor.

Dr. Archana:

Yeah, you can start.

Dr. Ronnie:

Good morning and a warm welcome to this focus group discussion session today. Myself, Dr. Ronnie from the Department of microbiology is an investigator of this project. And we have with us Dr. Archana from the Department of Medical Education who is also co-investigator in this project.

So this focus group discussion is conducted as part of the study titled '*The Perception of Online and Face-to-face Microbiology Laboratory Sessions among Medical Students and Faculty at Arabian Gulf University*.' Thank you all once again for giving your informed consent for taking part in this discussion. And please share your views about your

experiences with online as well as face-to-face microbiology laboratory sessions at AGU. So this study is approved by the research and ethics committee, AGU, number E049BI4/21. The discussion will be video recorded to ensure that we do not miss any of your valuable comments. So it would last for around 20 to 30 minutes, and all the contents of this discussion will be maintained confidential and your identity will not be revealed in any manner. The transcribed files will be kept in a secure location and will be destroyed after completion of the study. So please feel free to express your thoughts. And we will try our level best to take your valuable comments into account for our future microbiology laboratory sessions. Thank you all once again. So let's begin, over to you Dr. Archana.

Dr. Archana:
Thank you, Dr. Ronnie. First of all like this is a very good opportunity for all of you to express your views. Feel free to express your thoughts and suggestions. We are here to improve ourselves. You know it's - to err is human, we'd have done some mistakes, it is time for us to reflect on our experience and see what was done very nicely, what could have been done better and how are we going to take it forward. So students are very important stakeholders in the whole process. Of course, we will be taking input from faculty, we will take input from technical team and your input is very valuable as you are the end consumers of our product. So we value your opinion. So, let us start with our discussion.

I will be asking you around eight simple questions. The first three we will be focusing (0:10:00) mainly on online teaching and then fourth to sixth, the questions will be focusing on face-to-face lab sessions. And the last two ones are for your suggestions for improvement. So let me start with the first question. Can you please describe your experience with online microbiology lab session, your experience in general, about online microbiology lab sessions? Anyone can start, if someone wants to interfere or someone wants to continue with the comment, please go to reaction and raise your hand, we will come back to you. Yes, Zara, your mic was unmated, you want to say something?

Zara:
Yes, I liked it more than the face-to-face sessions.

Dr. Archana:
Can you explain more? What made you like it more?

Zara:

Because first of all, it is recorded, so we can re-watch it again, sometimes in a face-to-face sessions if I was late at the beginning, then I have to ask the students for example, for what have been missed or like this. And also because when we are seeing the microscopic pictures, the doctor can point on the thing he's explaining. But there in the lab we are watching the pictures by ourselves. So he can't point on what he's explaining. And also because the session time for in the face-to-face when sometimes the other group arrive, and the doctor still didn't continue his explanation, so we have to go to the other doctor to explain the others, the other. like, we have different doctors to explain different topics, right. So here we, in the online sessions the doctors have enough time to explain his part and all the students received the same information. And also, we have enough time to ask questions. So this made me feel that the online sessions are much better.

Dr. Archana:

Thank you, Sara. Looks like Madhavi also agree with Sara. Anyone else who want to contribute about your experience during online microbiology lab session? Yes, Sarah.

Sarah:

I will say my experience, I liked it as well more than the face-to-face sessions. As Zara said, it's recorded so we can go back to it again and again. The questions are documented, like the questions of the students, and the answers of the doctors are documented and recorded in the - and downloaded on Moodle so we can go back to it again. Also, the timing is very flexible. We used to wait for the other groups to finish outside the laboratory and then we wait for them to finish and then we go inside. And sometimes it's time consuming. So the timing of online microbiology labs were very flexible.

Dr. Archana:

Thank you, Sarah. Anyone else? Yes, Noah, proceed.

Noah:

I totally agree with Sara and ___. For me, the online sessions were very excellent as for many aspects, for example, the way the doctors have explained the material, it was more clear. Also, one problem we faced in the face-to-face sessions that the group size was large. So sometimes, some students they don't have space in the lab or for example, when the doctor would explain on something and for example, some experiments or on show it under

the microscope or on the culture, not all the students will see clearly. Also in the online teaching the doctors also have tried many ways how to explain the material for example, some videos were provided. That's it.

Dr. Archana:
Thank you, Noah, looks like Maria also agrees with Noah. Anyone else?

Noor:
Yeah. Everyone, like Sara and Noah have explained most of the issues (0:15:00) that we like about online learning. In addition to that, sometimes we have, like lecture, like lectures before -sessions before we go to the lab. And it's usually done in the hall. I forgot its name and because it's like, there is a projector, and everybody is at the same level, sometimes because I'm a short person, so I can't see clearly how are the slides and everything, because of the people who are in front of me. So, the online learning has helped me in that because the screen is just in front of me and the doctor can, like explain, and I can see ___.

Dr. Archana:
Thank you, Noor.

Noor:
Adding to what they said before because I agree with all of that.

Dr. Archana:
Thank you, thank you, Noor. Amina.

Amina:
I totally agree with my colleagues at the point that they ___ out, okay. And another thing in online ____ is better time management than face-to-face because before not only in microbiology lab, like even in other session, doctors sometimes they are late to their session, but in online session they are more on time rather than before because it's only like, they will only like press on the link and they will be there. But before no, they have to come from somewhere else, they maybe they have tutorial, they have something so sometimes they get late, but in online, no, there is better time management, even the doctor and microbiology lab, when ___ time the doctor will stay with the time, he will not take more time or less time, but better time management.

Dr. Archana:

Thank you, Amina. Now, let us go to the second question. I think some of you have answered it already, but still I want to make it explicit. In your opinion, what are the advantages of online microbiology lab session, just the advantages? What would you like to say these are the advantages of online microbiology lab session? Yes, Amina.

Amina:

As the girls said, the recorded lectures are the advantages of the online lecture. Sometimes maybe like from like weak connection, weak connection to the internet. So, some students will not be able to attend or not fully attend the lecture. But because of the recorded lecture, they will have the ability to attend and concentrate more.

Dr. Archana:

Thank you, Amina. Anyone else would like to add to the advantages of online microbiology lab session? Yes, Madhavi.

Madhavi:

Yes, in the face-to-face sessions sometimes we are divided into two groups to before the lecture and also before the explanation. So some group will get the full information and some others maybe the doctor will miss something. And the online session everyone will get the same information. And this is a good thing.

Dr. Archana:

Thank you. Thank you, Madhavi. Anyone? Anyone else who wants to say something about advantages of online microbiology lab session?

Maria:

I think it was more focused since it is just me and the doctor under ___ so there is no other students to make a distraction for me.

Dr. Archana:

Thank you, Maria. Now we go to the next question which brings to the other paradigm of the same domain. In your opinion, what are the disadvantages of or any difficulties you faced in online microbiology lab session, we're talking about lab, microbiology lab. So what

are the difficulties or disadvantages you think, no, this is not good with online session of microbiology lab? Are there anything? Can you please share?

Noah:

For me, despite the good points or the advantages of the online session, but I find it less motivating for discussion and even participating. Because we are sitting in the same room for the whole year, so I don't find it motivating for me (0:20:00) to participate, or sometimes even concentration is not. I'm not concentrating very well. Also, as, for example, under the microscope we have to see some slides, we are not able to see them in the online sessions, but some pictures will be provided by the doctors. But I believe that we should touch the microscope and see by ourselves.

Dr. Archana:

Thank you, Noah, thank you. Maria, your hand is up?

Maria:

Yes, I just want to say that for the practical part, not the theoretical part of the lab, we need to see real specimens under the microscope. We need that feeling and also we need to see the lab techniques, how it is performed, vaccine and other lab techniques.

Dr. Archana:

Thank you, Maria, Zara.

Zara:

Just to elaborate on what my colleagues said, the same points. It's not interesting. We don't interact very freely. We don't get involved in the procedure like the regular lab sessions especially the ___ as Maria said.

Dr. Archana:

Thank you. Thank you, Zara. So now we move towards the face-to-face microbiology lab session. What was your experience with face-to-face lab session microbiology? Describe your experience how you felt when you attended the face-to-face microbiology lab sessions?

Madhavi:

We will be more motivated.

Dr. Archana:

Thank you, Madhavi. Anyone? Zara, yes.

Zara:

A group of the attending students were a lot…

Dr. Archana:

Not able to hear you, Zara.

Zara:

___ the online - now, do you hear me?

Dr. Archana:

Yes, yes proceed.

Zara:

Now do you hear?

Dr. Archana:

Yeah, now it's better. Yeah.

Zara:

So there if we compare the attendance of the students in the online compared to the regular lab sessions, in the online, we only get maybe 16 students attending the online session, maybe it's not interesting to them. They don't want to attend it online. It's not fun. But in the regular lab sessions, we usually have more than half of the batch attending the lab sessions.

Dr. Archana:

Thank you, Zara. Anyone else wants to share anything about your experience of face-to-face lab session?

Sarah:

I have something, doctor. That when I attend in the real place, I can remember the things more easily. So for example, today, I can remember something from year three, because I attended by myself. So I think it is easier for me to remember things.

Noor:

I agree with Sarah.

Dr. Archana:

Can you …

Noor:

It's more personal, it's more personal. And it's on a personal level with the doctors that you ask them. Like, I remember asking Dr. Rohini some questions. And I still remember the species that I was asking about. So sometimes because we have the questions and we can have like one to one interaction with the doctors, it helps with the memory.

Dr. Archana:

Thank you, thank you, Noor. Yes, Zara.

Zara:

About the pictures under microscope for example, if we interpret the pictures by ourselves and by discussion with the other students, it helps a lot for memorizing, what are the findings and then in the ___ exams, we can easily do the questions and find what is the species, what is the bacteria and what can we find in this picture.

Dr. Archana:

Thank you. Thank you. Anyone else? Okay, now, can you list the advantages of face-to-face lab session? Just the advantages? Yes, Noor.

Noor:

I found it more motivating for a discussion, for asking questions, participating. And also one point I liked about the face-to-face that there were multiple (0:25:00) stations. So, different doctors would explain different material and so, I find it better. So, maybe I understand for one doctor better than the other. So, for me this has been efficient and there is also a gap between the two material, not with the online session, it is continuous.

Dr. Archana:

Thank you, thank you, Noor. Any more advantages of face-to-face?

Female Speaker:

I agree with Noor that every topic will be explained by other doctor because I can remember the doctor's voice with the material he gave us so it is easier for me to remember it.

Dr. Archana:

Thank you, Amina?

Amina:

Yes, the advantage of face-to-face that we are going to need to practice like how to, like how is the microscope, how to focus, how to put the slide. So, when we - we get older, I mean, someday may become a doctor, we still remember these techniques. But now unfortunately, we are unable to ___ because ___ because of ___. So this is really the advantage of face-to-face lecture was we have to use our hand and microscope and now we are going to be ___.

Dr. Archana:

Thank you, Amina. Amina. even your voice is not clear. Sometimes it is a little broken. But okay, we were able to understand what you said. Yes, Noor.

Noor:

I have one point to add, about the different doctors explained different material. I believe that when the doctor will explain the whole material, he will be over one. And sometimes he will forget some information to be added to the presentation. So when it is separated, or when it's divided to different doctors, the doctor will have sufficient time to explain about his material and will go deep into that.

Dr. Archana:

Thank you, thank you, Noor.

Noor:

Also, it's about the students itself, in the practical or face-to-face the students would be present for the whole session because he's there and he's interacting. While in the online, you can say that some can only like join the session and just say like without a video and doesn't interact, and maybe even doesn't even listen to the lecture itself.

Dr. Archana:
Understood, thank you. Amina, your hand is still raised, you want to say something?

Amina;
No, sorry, I will remove that.

Dr. Archana:
Okay, thank you. Now, the next question is, what are the disadvantages? What is that you didn't like about face-to-face lab session? Are there any disadvantages which you didn't like about face-to-face sessions? Yes, Amina.

Amina:
Yes. Sometimes it is time consuming not really the lecture itself but sometimes some people like their houses is away from university, but when they come to the university, it will take time then they will stay at university for the lecture. Then from lecture, maybe it is late. So they will stay at university like to study. So I think in general face-to-face lectures, sometimes they could be time consuming. They take a lot of time from our day.

Dr. Archana:
Okay, thank you, Amina. Zara.

Zara:
One thing I didn't like about the microbiology labs, the face-to-face sessions is that we were divided into two groups, group A and group B. And, for example, if I'm in Group B and Group A starts, like the doctors don't put very specific or accurate time for the lab sessions. So we used to wait for 45 minutes outside and then we wait for the other group to finish. So it would be better if they specify accurately at which time the lab ends, so no one (0:30:00) has to wait for the other group to finish.

Dr. Archana:

Thank you. Thank you, Zara. I saw someone else also wanted to say something.

Madhavi:

Yes, doctor, I want to say something.

Dr. Archana:

Yes.

Madhavi:

It's the same as what Noah says that we are divided into groups. And in the practical parts, we have to make like a smaller group, like a circle, and some students cannot say, so they will be in the back. So they cannot see all the session. So this is not good thing about a face-to-face session.

Dr. Archana:

Thank you. Anyone else who wants to add disadvantages, or limitations of face-to-face lab session? Yes, Noor.

Noor:

Also one point I think that the girls already mentioned. But I want to say that the face-to-face sessions we had no recorded videos for the lectures. So whenever I forget some information, I want to go back for it. I don't have the video record. So this was a little bit hard part.

Zara:

I have also something like that.

Dr. Archana:

Yes, Zara.

Zara:

At the end of the session, at the end of the time when the other group arrive the Dr. Harry's lab, and he didn't - he doesn't finish the material, because he's waiting for the other group or they are waiting. So the information for both groups can be different sometimes.

Dr. Archana:

Thank you. Thank you, Zara. Anyone else? Okay, now we have heard arguments for both face-to-face and online session. Now, the next question is, do you think blended learning, you know, what is blended learning? It is a combination of online teaching with face-to-face. So we take the best of both the worlds, that is called blended learning. Do you think a blended learning would suit microbiology lab session? If yes, why do you think? Yes, Maria.

Maria:

I think it will be beneficial for the theoretical part or if we have cases to solve, with no practical part it is better to be online, or if there is practical part. I think it is better to be face-to-face so we can get useful of the blended learning.

Dr. Archana:

Thank you, Maria. Noah, yes.

Noah:

The answer is yes because sometimes we need to see the slides under the microscope or some techniques in the lab. And so for me, I prefer the face-to-face learning as I find it better for my learning and for my memory. So I can perform and see by myself. And ask different doctors different questions. Because in the online session, there is only one doctor, I can ask him my questions, but in the face-to-face I can go to other doctors. But I believe that the pure scientific material doesn't need personnel attendance. So the online sessions will be enough. So this is how the combined or blended sessions will be done.

Dr. Archana:

Thank you. Thank you, Noah. Amina, you want to say something?

Amina:

Yes. So as Noah said, certain things need to be face-to-face, but maybe like other things we can - we could use online like sometimes, like when we put a case with this method, myself, I prefer to be online because everyone will see clearly the case. Everyone maybe will have it, not everyone but people will have and also debate ___. Because before we sit in a hall and the girls already said that sometimes it is hard to see the board. But when we put it online, everyone will be able to see the case and maybe discuss it. But in the particle side, that's a face-to-face.

Dr. Archana:

Thank you, Amina. Anyone else?

Noor:

I mean, I would add to what they said. Like most of us has the same understanding that online learning is better for the long sessions that only need a presentation without like only information, pure information (0:35:00) and theoretical part, while for the face-to-face, it's better for the, if we have a lab session like we would see, we would use the microscope, or we would see a particular like, lab technique or something. With that, I think also that the face-to-face has its advantages with memory, but also for the online sometimes I remember, we had like a long session, that's, it's tiring, sometimes the face-to-face. So the online learning helps with that. So overall, the blended learning would be a much better fit for me, as of – if it's either or the both.

Dr. Archana:

Thank you. Noor, thank you. Thank you, all of you. Now we have our last question.

Female Speaker 2:

Sorry, ___.

Dr. Archana:

Sorry, Noah, proceed, proceed.

Noah:

Foe me maybe the perfect way to combine both sessions is when there is the scientific material, the doctors will teach us by online sessions. And the next day, we can attend personally to see what the doctor has explained. For example, see under the microscope or some techniques to be seen. So it will save time for both the doctor and the students. And, like we repeat also on the material, so it's better for our memory also.

Dr. Archana:

Thank you. So you're meaning to say one day of theory with online following which the next day could be practical where you can revise what was taught in the theory and you save time, this is what?

Noah:

Yes.

Dr. Archana:

Okay. Thank you, Noah, yes Zara.

Zara:

I agree with Noah.

Dr. Archana:

Thank you. Amina, your hand is still raised, you want to say something?

Amina:

No, sorry.

Dr. Archana:

Okay, no problem. Okay, this brings us to the last question. There are few topics in microbiology lab session, you know, like sample handling, processing, recording and interpretation of the results. So, I think Dr. Ronnie will be a better person to explain, we want to know your opinion whether you want these sessions to be online or face-to-face? I repeat sample handling, sample processing, recording and interpretation of the results. Yes, Zara.

Zara:

I prefer it to be face-to-face.

Dr. Archana:

Can you - can you be louder?

Zara:

I prefer it to be face-to-face.

Dr. Archana:

Okay, thank you, Maria.

Maria:

Yes, I think it would be better if it is face-to-face. But all the students can see like there is documentation for it also for other students who has some information.

Dr. Archana:

Okay. Ronnie, do you want to add something to this last query?

Dr. Ronnie:

I think they have understood, right, the sample processing how would we do, the techniques what we use or the tests what we do. So all those whether you need face-to-face or it will be better if it is online.

Zara:

For me doctor, it is better face-to-face for the practical part because even when they shared a video it was very slow in the online and it was breaking. So it is better if it was face-to-face for the practical part.

Dr. Archana:

Thank you, Zara. Amina.

Amina:

Yeah, ___ we cannot choose both like ___ and with blended, face-to-face and the online.

Dr. Archana:

For the …

Amina:

We can choose both or only one?

Dr. Archana:

Ronnie, she is asking for these topics can we choose blended learning? That's what she's asking, combination of both, theory in online and only the practical component face-to-face.

Amina:

Yes, the practical component (0:40:00) is face-to-face of course. The practical part is face-to-face. Okay. And but as I said before the cases yeah, I for myself, I prefer it to be online because it will be well documented. Okay, and everyone will be able to see it because before we used to do in a hall, very big hall, a lot of people sit behind because maybe they are late, maybe because of traffic, I don't know. So they are not able to see. So it's better to be online. Thank you, doctors.

Dr. Archana:

Thank you. Thank you, Amina. That's all from our side. Anything else you want to add which is not covered in these questions? Anything you want to let us know? Anything you want to share. Yes, Noor.

Noor:

Sorry I disconnected and I reconnected again. But for the I prefer the face-to-face or the online, I'd go for the face-to-face.

Dr. Archana:

Okay, for all the sessions. You mean?

Noor:

I will for all the sessions or the sample handling.

Dr. Archana:

Okay. Okay. agreed. Thank you. Anyone else from your side, which is not covered in these questions, but you want to share from your experience because you have been through both methods of training, anything else you want us to take into consideration? Any suggestions, feedback? Yes, Zara.

Zara:

Maybe. For me, if it was like even if online session, different topics with different doctors, like not only one doctor that will explain all the topic. For me, it will be better.

Dr. Archana:

Thank you.

Dr. Ronnie:

So you mean like the stations what we used to have the same way do it online with two, three doctors or how many ever doctors are?

Zara:

Yes, we have this in the malaria problem. This unit, Dr. Abdul Rahman explained the life cycle and Dr. Mohammed Shahid explained all..

Dr. Archana:

___.

Zara:

Yeah. So it was for me good.

Dr. Archana:

Thank you. Thank you. Any other suggestion? Noor.

Noor:

Yes, for the future, I find that maybe it's better to stick with the face-to-face. And the students already you have, they have the recorded online sessions because all the year two, year three, year four they have - they had the online session. So they already have the recorded sessions, whenever they want they can go for them. But for me, I find that the old way is better.

Dr. Archana:

Thank you, Noor. Anything else?

Maria:

I just want to reemphasize on back to the theoretical part will be a day earlier than the practical part. So the students will have time to study and have an idea before seeing video link and applying what they know.

Dr. Archana:

Okay, thank you. Just I want to add one more thing since it was not brought up in the discussion. Do you think that social presence or social interaction with your friends, the

communication with your friends was it better in online or in face-to-face? You know the presence of your friends? Yes, Zara.

Zara:
I think the presence of us with our friends and interacting with them is very important. Like I can still remember things from year two and three but I can hardly remember things from year four what I've just studied. I don't know, maybe because it's online. And I can just repeat it on myself and study by myself but when interacting with other people they can raise questions, ideas on how to memorize things and maybe say this looks like ___ or on the specimen or something and I can remember it forever. But in the online, I study just by myself and I have just my ideas and my thoughts and no one is interacting with me. So it makes it more difficult to memorize things for the long term.

Dr. Archana:
Thank you, Zara. Thank you, anyone else? Okay, so can we conclude the session? Ronnie, you want to add something?

Dr. Ronnie:
No, no, doctor, thank you so much. And I would like to thank all my students for participating in this group discussion. And, sure, we will consider all your feedback, and we'll try to improve our microbiology laboratory sessions.

Dr. Archana:
Thank you guys. Thank you very much. It was a learning experience for us also. You have given very good valuable suggestions, we'll try our best to implement as much as possible, things which we cannot implement we will write it as a recommendation to the concerned authorities and we will try to make it possible at the earliest. Thank you so much. Thank you for your time. Wish you all the best. Thank you,

Chorus;
Thank you, doctor.

Dr. Archana:
Bye-bye.
